# Binding Affinity of Synthetic Cannabinoids to Human Serum Albumin: Site Characterization and Interaction Insights

**DOI:** 10.3390/ph18040581

**Published:** 2025-04-16

**Authors:** Rita M. G. Santos, Rita Lima, Sara Cravo, Pedro Alexandrino Fernandes, Fernando Remião, Carla Fernandes

**Affiliations:** 1 Laboratório de Química Orgânica e Farmacêutica, Departamento de Ciências Químicas, Faculdade de Farmácia, Universidade do Porto, Rua Jorge Viterbo Ferreira, 228, 4050-313 Porto, Portugal; anaritasantos7890@gmail.com (R.M.G.S.); ritaalexandralima@gmail.com (R.L.); scravo@ff.up.pt (S.C.); 2 Interdisciplinary Center for Marine and Environmental Research (CIIMAR), University of Porto, Terminal de Cruzeiros do Porto de Leixões, Avenida General Norton de Matos, 4450-208 Matosinhos, Portugal; 3 LAQV, REQUIMTE, Departamento de Química e Bioquímica, Faculdade de Ciências, Universidade do Porto, Rua do Campo Alegre, S/N, 4169-007 Porto, Portugal; pafernan@fc.up.pt; 4 UCIBIO—Applied Molecular Biosciences Unit, Laboratory of Toxicology, Department of Biological Sciences, Faculty of Pharmacy, University of Porto, Rua Jorge Viterbo Ferreira, 228, 4050-313 Porto, Portugal; remiao@ff.up.pt

**Keywords:** binding affinity, docking, displacement studies, HPAC, HSA, synthetic cannabinoids

## Abstract

**Background/Objectives:** High-performance affinity chromatography (HPAC) was used to investigate the binding affinity of a series of synthetic cannabinoids, a widely abused class of new psychoactive substances, to human serum albumin (HSA) and obtain insights into the binding sites. To better understand the recognition mechanisms, molecular docking studies were conducted. **Methods:** Binding affinity was assessed through zonal elution approach Additionally, displacement chromatography with site-specific probes provided insights into the HSA binding sites of five synthetic cannabinoids. **Results:** That these drugs exhibit extensive binding to HSA, with values ranging from 98.7% to 99.9%. Competition for site I was observed between warfarin and four synthetic cannabinoids (5F-AMB, AB-PINACA, AMB-FUBINACA, and AB-CHMINACA). Furthermore, AB-CHMINACA also competed with L-tryptophan for site II. The binding affinity of all synthetic cannabinoids increased in the presence of (*S*)-ibuprofen. Molecular docking studies supported the experimental findings, reinforcing the insights gained. **Conclusions:** The key novelty of this study lies in analyzing, for the first time, the binding affinity of synthetic cannabinoids to HSA through HPAC and molecular docking. These results may improve our understanding of their toxicokinetic behavior and help in predicting possible competitive interactions that could influence HSA binding and, consequently, their activity and toxicity. This study is the first to describe the binding affinity of synthetic cannabinoids to HSA, elucidate their recognition mechanisms, identify binding sites, and characterize their interactions with the protein.

## 1. Introduction

Synthetic cannabinoids are a chemically and structurally diverse group of new psychoactive substances (NPS) designed to target the endocannabinoid system. These NPS exhibit a significantly higher affinity for cannabinoid receptors (CBRs) than Δ^9^-tetrahydrocannabinol (Δ^9^-THC), the primary psychoactive component of cannabis. As a result, they may produce more intense psychoactive effects, along with more severe adverse effects [1,2]. Consumers of these drugs typically seek their known psychoactive effects, such as relaxation and euphoria. However, harmful effects can emerge rapidly due to various factors, including the formation of toxic metabolites, drug interactions, dosage, and more. Additionally, chronic exposure can alter neuronal structure, impair cell viability, and cause DNA damage [3,4].

Synthetic cannabinoids first emerged between the 1960s and the 1970s, when researchers began exploring how the endocannabinoid system modulates biological functions [1,5]. Today, these drugs dominate the NPS market [6]. The rapid emergence of these compounds raises significant concerns related to their biological and toxicological effects, as well as the risk of inadvertent consumer exposure, given the growing availability of adulterated products that frequently escape detection [6,7].

These NPS are primarily consumed by inhaling plant material infused with the substances, commonly known as “herbal smoking mixtures”, or through e-liquids in electronic vapes [8]. This mode of administration allows for rapid absorption through the alveoli and swift redistribution to other organs, including the brain, reaching maximum concentrations within minutes of consumption [1,4]. Notably, due to their lipophilic nature, synthetic cannabinoids are expected to bind extensively to plasma proteins [9], leading to high volumes of distribution. In vitro pharmacokinetic studies on a series of synthetic cannabinoids, plasma protein binding affinity was assessed using equilibrium dialysis. The results showed a high degree of protein binding, ranging from 88.9% to 99.9% [10,11]. Moreover, for the synthetic cannabinoid WIN 55,212-2, the plasma protein binding was 95%, measured using ultrafiltration and subsequent analysis with LC-MS [12].

During distribution, drug binding to plasma proteins is a reversible process that exists in an equilibrium between bound and free fractions. Only the free fraction can cross membrane barriers and reach biological targets [13,14]. Therefore, monitoring a drug’s binding affinity to plasma proteins is essential for understanding its behavior in the body and determining its overall biological/toxicological profile [15,16]. Notably, when a drug with a high plasma protein affinity is administered at high concentrations, binding saturation may occur. This leads to an increase in the free drug fraction in the bloodstream, which can influence both biological activity and toxicity [17]. Additionally, competition between two drugs or between a drug and an endogenous molecule or dietary compound can alter protein binding, further affecting the free drug fraction [18,19].

Studies have shown that 43% of the 1500 most commonly used therapeutic drugs interact with plasma proteins [20], often displaying a high affinity for human serum albumin (HSA) [21]. HSA is the most abundant plasma protein and plays a crucial role in transporting various endogenous compounds, drugs, and metabolites [22,23].

Several methods have been described to study drug binding to HSA [24,25,26]. Among them, high-performance affinity chromatography (HPAC) has proven to be a highly effective and efficient technique for analyzing intermolecular interactions between HSA and drugs [27]. HPAC can also be used for displacement studies to determine the specific binding sites of drugs on HSA [28,29]. Another valuable approach for analyzing HSA binding sites and understanding recognition mechanisms is using in silico methods, such as molecular docking [30]. Docking studies enable the atomic-level simulation of drug–HSA interactions, providing insights into the drug’s behavior within the target protein binding site [31,32].

**Figure 1:**
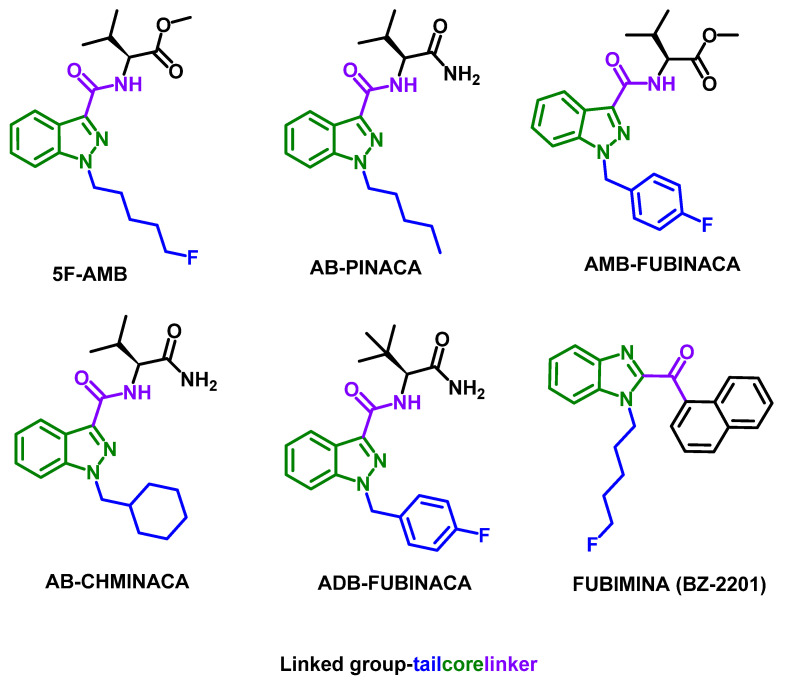
Chemical structures of the synthetic cannabinoids.

Herein, the evaluation of the binding affinity of six synthetic cannabinoids (Figure 1) to HSA is described. The library comprises five indazole carboxamides, 5F-AMB, AB-PINACA, AMB-FUBINACA, AB-CHMINACA, and ADB-FUBINACA, and one benzimidazole, FUBIMINA (BZ-2201). Structurally, they consist of a “core”, a “linker”, a “linked group”, and a “tail” (Figure 1). For the indazole carboxamides, displacement experiments with well-known probes were carried out to obtain information on the specific sites that drugs bind to HSA. Both studies were performed with HPAC using an HSA-based column [33,34]. Additionally, docking studies were performed to analyze the binding sites and better understand the interaction mechanisms between the synthetic cannabinoids and the protein.

To the best of our knowledge, no studies in the literature have yet described the binding affinity of synthetic cannabinoids to HSA. Additionally, no reports were found on their interaction sites or recognition mechanisms. Therefore, the key novelty of this work lies in analyzing, for the first time, the binding affinity of a series of synthetic cannabinoids to HSA using HPAC and molecular docking. This study aims to elucidate their recognition mechanisms, identify binding sites, and characterize interactions with the protein. These findings could enhance our understanding of their toxicokinetics and help predict potential competition that may affect HSA binding, ultimately influencing their activity and toxicity.

## 2. Results and Discussion

### 2.1. Determination of HSA Binding Affinity

Binding affinity studies were conducted using a series of commercially available synthetic cannabinoids (Figure 1). Despite their commercial origin, the structures of these compounds were confirmed through spectroscopic and spectrometric techniques, with the corresponding data and spectra provided in the Supplementary Materials. The binding affinity of the synthetic cannabinoids to HSA was measured based on previously established methods using HPAC [15,35,36]. For this purpose, a commercially available HSA column, CHIRALPAK^®^ HSA, was employed. The analyses were performed using zonal elution in reversed-phase mode. A potassium phosphate buffer solution (67 mM, pH 7.0) was selected as the mobile phase due to HSA’s stability in phosphate buffers [37], and to closely mimic physiological conditions [38,39]. Several studies describe the use of this buffer in protein binding studies mimicking the physiological conditions of human plasma [19,28,31,38,39].

Given the high retention of the compounds on the HSA column, an organic solvent was added to the aqueous mobile phase. ACN was chosen for the HSA studies, as it is one of the most commonly used organic modifiers in HSA columns [26]. However, a low percentage of the organic modifier was necessary to prevent protein denaturation and prolong the column’s lifespan [40]. Additionally, the presence of an organic modifier must be carefully considered, as it can alter the number of available binding sites by affecting the spatial conformation of the protein [41].

To calculate the bound fraction (%*b*) to HSA, different proportions of organic modifier were added to the mobile phase, ranging from 11% to 15%. In the Supplementary Materials (Figures S1–S6), representative chromatograms for each synthetic cannabinoid are shown, as examples. Due to the higher retention of FUBIMINA, a higher percentage of ACN was required, reaching up to 16%. All analyses were performed in triplicate. As the percentage of the organic modifier increased, the retention times of the synthetic cannabinoids decreased (Figure S7, Supplementary Materials). To determine the %*b* values in a 100% aqueous buffer, an extrapolation was performed by plotting the logarithm of the retention factor (log *k*) against the percentage of the organic modifier [31] (Figure S7, Supplementary Materials).

According to Equation (4), the %*b* values were calculated and are presented in Table 1. Three compounds, chlorpromazine, indomethacin, and metronidazole, were used as controls, as their %*b* values to HSA have already been reported [42,43,44]. To determine the experimental %*b* values for these controls, different proportions of ACN were added to the mobile phase, ranging from 2% to 20%. The experimental %*b* values showed good agreement with the reported values: 82.12% for chlorpromazine (reported: >90% [43]), 96.89% for indomethacin (reported: 97.8% [42]), and 19.78% for metronidazole (reported: 20% [44]).

As illustrated in Table 1, all synthetic cannabinoids exhibited very high affinity for HSA, with %*b* values ranging from 98.7% to 99.9%. As expected, FUBIMINA presented the highest binding affinity to HSA, with a %*b* value of 99.9%

It is known that HSA presents high affinity for lower molecular weight, lipophilic, and negatively charged compounds [19,28,42]. Although the synthetic cannabinoids are not negatively charged, they are lipophilic molecules, as evidenced by their Log P_o/w_ values (Table 1). This pronounced lipophilicity is expected to promote strong interaction with the protein, resulting in high binding affinity.

Notably, the synthetic cannabinoid with the highest binding percentage to HSA, FUBIMINA, is also the most lipophilic compound, with a Log P_o/w_ value of 3.67. Similarly, AB-PINACA, the least lipophilic synthetic cannabinoid of this series, has a Log P_o/w_ of 2.58 and exhibits the lowest binding affinity. These findings suggest a correlation between the compounds’ lipophilicity and their binding affinity toward the protein.

### 2.2. Displacement Studies

The crystallographic structure reveals that HSA has a flexible structure consisting of three homologous α-helical domains (I–III). Each domain contains ten helices, which are further divided into two subdomains, A and B [45]. The primary binding sites for most endogenous and exogenous compounds are predominantly located within the hydrophobic cavities of subdomain IIA (site I) and subdomain IIIA (site II) [46].

To gain deeper insights into HSA binding, displacement studies were conducted to further investigate the binding sites of the synthetic cannabinoids on this protein. A zonal displacement chromatography approach was selected [26], involving the addition of increasing concentrations of three site-specific probes to the mobile phase: warfarin, which binds to site I of HSA [47], and (*S*)-ibuprofen and L-tryptophan, which bind to site II of HSA [22]. These competitors are commonly used in displacement studies to elucidate binding interactions at specific HSA sites [48,49,50,51,52].

Various concentrations of the competitors were added to the mobile phase, based on previous studies [16,19,28]. Given the high binding affinity of synthetic cannabinoids to HSA and the need for efficient analysis without excessively prolonging run times, an organic modifier (ACN) was added to the aqueous buffer and used as the mobile phase. Consequently, 13% was identified as the optimal organic modifier concentration for these studies. FUBIMINA was not considered for this study due to its high retention on the HSA column, making analysis time unfeasible under these conditions. A higher concentration of ACN in the mobile phase would be required, which could negatively impact the HSA column [40].

**Figure 2:**
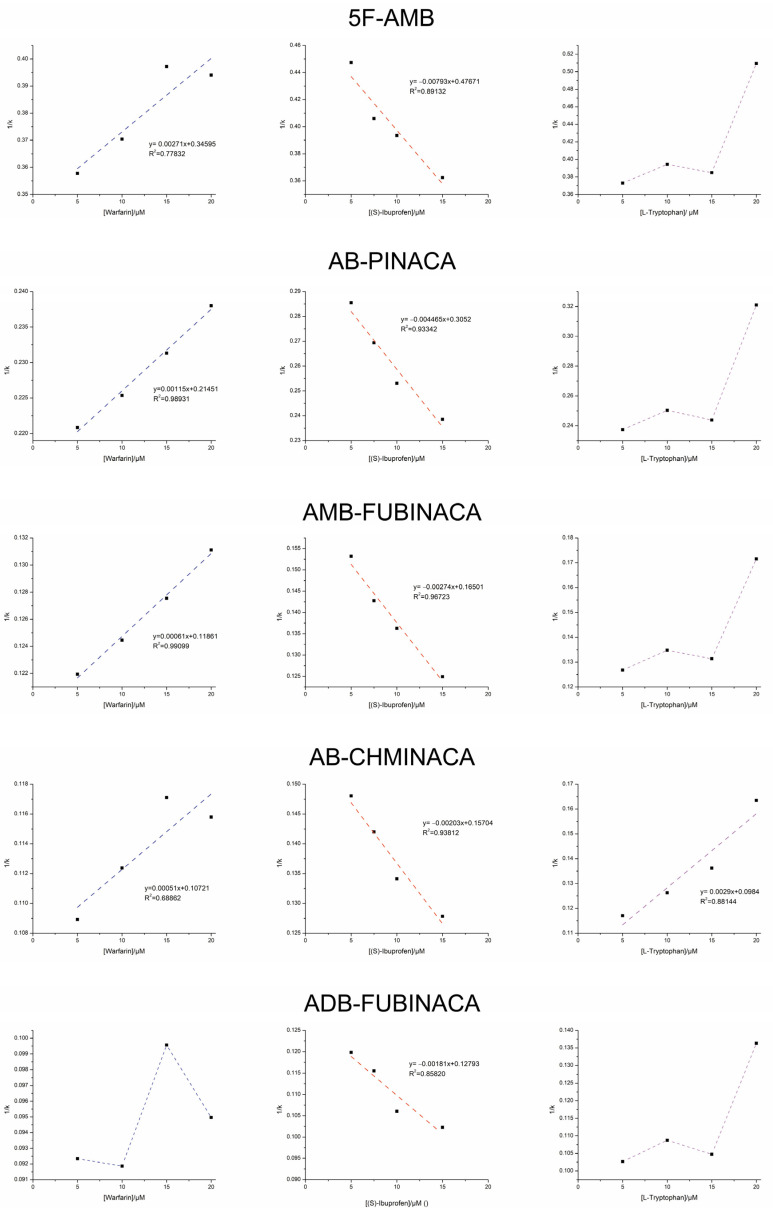
Displacement chromatography experiments with synthetic cannabinoids, in the presence of increasing concentrations of competitor, expressed as a plot of 1/*k* of the analyte versus competitor concentration.

The displacements of the analyzed synthetic cannabinoids in the presence of increasing competitor concentrations are summarized in Figure 2. The chromatographic data are plotted as 1/*k* versus the competitor concentration.

As shown in Figure 2, a decrease in *k* values with increasing warfarin concentrations was observed for all synthetic cannabinoids except ADB-FUBINACA, which exhibited random variations. Moreover, a linear relationship with a positive slope is observed in the plot of 1/*k* versus warfarin concentration. This suggests that the studied synthetic cannabinoids, except for ADB-FUBINACA, compete with warfarin for binding site I [28].

When comparing the structures of the synthetic cannabinoids, ADB-FUBINACA is the only one that contains a *tert*-butyl group in the linked portion of the molecule instead of an isopropyl group (Figure 1). The additional methyl group in ADB-FUBINACA may cause steric hindrance, preventing interaction with binding site I of HSA.

Surprisingly, when using (*S*)-ibuprofen, an increase in *k* values for synthetic cannabinoids was observed with increasing competitor concentration. Similar to warfarin, a linear relationship was found in the plot of 1/*k* versus (*S*)-ibuprofen concentration for all synthetic cannabinoids. However, in this case, the slope was negative. This result suggests that the studied compounds interact allosterically with this competitor [27]. The interaction of (*S*)-ibuprofen with HSA likely induces conformational changes in the protein, enhancing the binding affinity of synthetic cannabinoids at their interaction site.

To confirm that synthetic cannabinoids, despite competing with (*S*)-ibuprofen, do not effectively bind to site II, an additional displacement study was conducted using L-tryptophan as a site II-specific probe. For all analytes except AB-CHMINACA, the plot of 1/*k* versus L-tryptophan concentration exhibits random variations. This suggests that synthetic cannabinoids do not interact with site II, with the exception of AB-CHMINACA, which directly competes with L-tryptophan for this site.

In summary, 5F-AMB, AB-PINACA, and AMB-FUBINACA exhibit similar HSA binding behavior, as they compete with warfarin for site I, interact allosterically with (*S*)-ibuprofen, and do not compete with L-tryptophan. These findings indicate that they primarily bind to site I.

These findings offer valuable insights into potential competition affecting HSA binding, as several compounds interact with site I of HSA [53]. Among them are other NPS, such as synthetic cathinones [28], and hallucinogenic substances, including “Purple Drank” [19], which are often consumed together. When taken simultaneously and in uncontrolled doses, they can pose serious risks. Competition for HSA binding could increase the unbound fraction of synthetic cannabinoids or other drugs in the bloodstream, potentially leading to heightened side effects, increased toxicity, or even overdose.

Furthermore, in the presence of (*S*)-ibuprofen, these compounds are more effectively retained by the protein. This suggests that their binding affinity to HSA increases, resulting in a lower free fraction of these compounds, which could theoretically lead to a reduced biological effect.

AB-CHMINACA was found to bind to both site I and site II of HSA, as it competes with warfarin and L-tryptophan for these binding sites, respectively. Additionally, this compound exhibits allosteric interactions with (*S*)-ibuprofen. In contrast, ADB-FUBINACA was the only synthetic cannabinoid that did not show competition with the tested competitors, indicating that neither site I nor site II is its primary binding site in HSA.

Table 2 summarizes the binding behavior for each synthetic cannabinoid at the HSA sites.

*K_I_* can be used to determine the type of competition between an analyte and a competitor. If *K_I_* values are similar to the affinity constants of the competitors reported in the literature, direct competition between the analytes and competitors should be considered [54]. The *K_I_* values were calculated using Equation (6) and are presented in Table 3. These values were then compared with the reported affinity constants of warfarin, (*S*)-ibuprofen, and L-tryptophan found in literature [55]. In this study, the calculated *K_I_* values are significantly lower than those reported for the competitors, particularly for (*S*)-ibuprofen. This finding suggests allosteric competition between the analytes and competitors [54].

### 2.3. Docking Studies

In silico studies with the target, such as molecular docking, are crucial for gaining a deeper understanding of analyte–target interactions and characterizing recognition mechanisms [19]. Displacement experiments indicated that all the synthetic cannabinoids studied bound to site I of HSA, except for AB-CHMINACA. To further confirm the binding sites of 5F-AMB, AB-PINACA, AMB-FUBINACA, and AB-CHMINACA, molecular modelling and docking calculations were performed. These analyses aimed to elucidate the recognition mechanisms underlying the experimental results and identify the interactions between the synthetic cannabinoids and the protein. Synthetic cannabinoids were docked to the rigid HSA receptor using AutoDock Vina with an exhaustiveness parameter of 8. The docking focused on site I of HSA, with the grid box centered at X: 5.00 X: 5.00, Y: −8.00 Y: −8.00, Z: 7.00, and dimensions X: 20.6, Y: 17.6, Z: 18.0 Å. Up to nine conformations with the lowest binding affinities were analyzed for detailed interaction studies.

The docking scores for synthetic cannabinoids are presented in Table 4. A more negative docking score indicates a more stable protein–analyte complex [19]. In AutoDock Vina, this score represents the estimated binding free energy.

The results indicate that all synthetic cannabinoids exhibit significant affinity for site I of HSA, with binding free energies ranging from −8.2 to −9.6 kcal/mol. These values are comparable to those of warfarin, a site I-specific probe, which has a binding free energy of −9.8 kcal/mol [56]. A strong correlation (80%) was observed between the docking free energies and the experimental HPAC displacement data regarding the HSA binding site.

**Figure 3:**
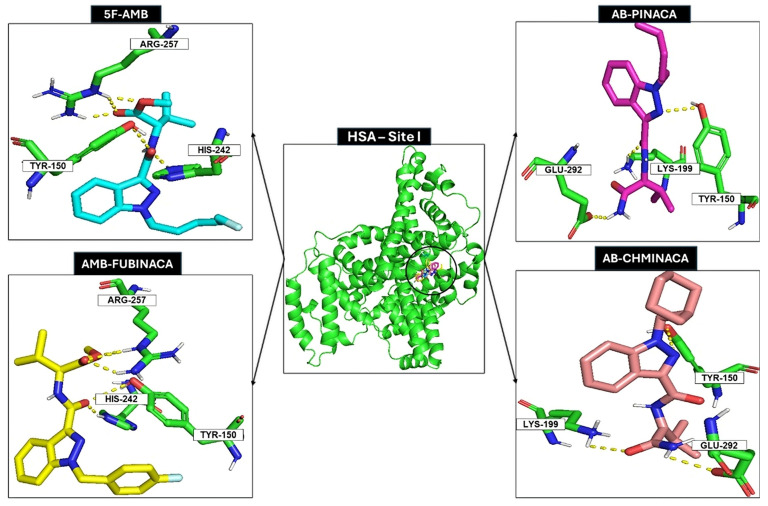
5F-AMB, AB-PINACA, AMB-FUBINACA, and AB-CHMINACA docking with HSA (site I). Nitrogen, oxygen, hydrogen, and fluorine are represented as blue, red, white, and baby-blue sticks, respectively. HSA (site I) is represented as a green cartoon, and 5F-AMB, AB-PINACA, AMB-FUBINACA, and AB-CHMINACA are represented as cyan, magenta, yellow, and baby-pink sticks, respectively. Hydrogen bond interactions between 5F-AMB and Tyr150, Arg257 and His242, AB-PINACA and Lys199, Tyr150 and Glu292, AMB-FUBINACA and His242, Tyr150, Arg257, AB-CHMINACA and Lys199, and Glu292 and Tyr150 are represented as yellow dashes.

The binding conformations of all the analytes at site I of HSA were visually inspected to interpret the binding free energies. Site I features a pocket primarily composed of hydrophobic and positively charged residues, proving a versatile environment capable of accommodating a diverse array of compounds [16]. It is mostly formed from a hydrophobic cleft composed of Tyr150, Phe211, Trp214, Leu219, Ala215, Phe223, Leu234, Leu239, His242, Leu243, Leu260, Ile264, Ile290, Ala291, and three residues, Tyr150, Arg222, and Arg257, that can establish polar contacts. Figure 3 illustrates representative examples of the best binding poses for each HSA–analyte complex at site I.

The amide group (linker) and the ester group (linked group) of 5F-AMB establish polar interactions/hydrogen bonds with residues His242 and Tyr150 and residue Arg257, respectively. The indazole group (core) and the carboxamide (linker) of AB-CHMINACA establish polar interactions/hydrogen bonds with residues Glu292 and Lys199, and with residue Tyr150, respectively. In terms of AB-PINACA, the indazole group (core) establishes polar interactions/hydrogen bonds with the residue Tyr150, the amide (linker) establishes polar interactions/hydrogen bonds with the residue Lys199, and the carboxamide (linked group) establishes polar interactions/hydrogen bonds with the residue Glu292. The amide group (linker) of AMB-FUBINACA establishes polar interactions/hydrogen bonds with the residues Tyr150 and His242, and the ester group, within the linked group, establishes polar interactions/hydrogen bonds with the residue Arg257. Finally, for ADB-FUBINACA, the amide group (linker) and the carboxamide group (linked group) establish polar interactions/hydrogen bonds with the residues Arg257 and Tyr150, respectively.

Despite all synthetic compounds exhibiting a general structure composed of a “core”, a “linker”, a “linked group”, and a “tail” (Figure 1), they present distinct structural features that lead to differential interactions with the protein. Notably, all compounds establish interactions with the amino acid residue Tyr150, albeit through different molecular moieties (Figure 3).

Except for ADB-FUBINACA, all the synthetic cannabinoids demonstrate strong binding affinity to HSA, corroborating the experimental results.

## 3. Materials and Methods

### 3.1. Chemicals

The synthetic cannabinoids 5F-AMB, AB-PINACA, AMB-FUBINACA, AB-CHMINACA, ADB-FUBINACA, and FUBIMINA were obtained from TicTac Communications Ltd. (London, UK) (https://www.tictac.org.uk). The structures of all synthetic cannabinoids were confirmed by IR, ^1^H NMR, ^13^C NMR, UV, and HRMS (data and spectra are shown in the Supplementary Materials, Figures S8–S26). Monobasic potassium phosphate (99% pure) and dipotassium hydrogen phosphate (99% pure) were of analytical grade. Acetonitrile (ACN), ethanol (EtOH), and 2-propanol (2-PrOH), for HPLC, were purchased from Sigma-Aldrich Co. (St. Louis, MO, USA) along with warfarin (98% pure) and (*S*)-ibuprofen (99% pure). *L*-Tryptophan (99% pure) was obtained from Thermo Fisher Scientific (Waltham, MA, USA). The ultrapure water was generated with a Milli-Q system, conductivity ≤ 0.1 µS cm^−1^ (Millipore, Bedford, MA, USA).

### 3.2. Instrumental and Chromatographic Conditions

The chromatographic analyses were performed in the UHPLC Dionex UltiMate 3000 system (Thermo Fisher Scientifc Inc., Waltham, MA, USA), equipped with a 3000 quaternary pump, autosampler, and variable wavelength detector. Chromeleon^TM^ 7.2 Ultimate (Thermo Fisher Scientifc Inc., Waltham, MA, USA) was the software used to process the chromatographic data. The Chromaster HPLC (HITACHI, Ltd., Chiyoda, Tokyo, Japan) equipment, complete with a 5160 quaternary gradient pump, 5260 standard loop autosampler, a 5310 column oven, and a 5430 DAD, was also used. The software used to process the chromatographic data was Clarity VATM 8.6.0.48 (Data Apex, Inc, Prague, Czech Republic).

The chromatographic analyses were performed using a CHIRALPAK^®^ HSA column (150 × 40 mm I.D., 5 µm particle size) from Chiral Technologies Europe (Daicel Chemical Industries, Ltd., Osaka, Japan). The sample injections (10 µL) were carried out in triplicate, and the flow rate was set to 0.5 mL/min.

The analyses were performed in reversed-phase mode using as mobile phases mixtures of 67 mM potassium phosphate buffer solution (pH 7.0) with different proportions of ACN. For HSA binding affinity studies, ACN proportions ranged from 11% to 16%, whereas a fixed proportion of 13% ACN was used for displacement studies. All chromatographic analyses were performed at 25 ± 2 °C, under isocratic mode, with UV detection at 220, 254, and 300 nm. All mobile phases were prepared in a volume/volume ratio (*v*/*v*), filtered through polyamide membrane filters of 0.2 µm pore size from Whatman^®^ GmbH (Dassel, Germany), and degassed in an ultrasonic bath (Soltec^®^ Sonica^®^ Ultrasonic cleaner) for at least 15 min before use.

Warfarin, as a site-specific probe for Sudlow site I, and L-tryptophan and (*S*)-ibuprofen, as site-specific probes for Sudlow site II, were employed for displacement studies [46].

### 3.3. Samples and Buffer Preparation

Stock solutions of all synthetic cannabinoids were prepared in EtOH at the concentration of 0.2 mg/mL and further diluted with the mobile phase, the potassium phosphate buffer (67 mM, pH 7.0), to a final concentration of 50 μg/mL. All working solutions were filtered through a membrane of 0.45 µm pore size and vortexed for at least 30 s before injection. The probe solutions of warfarin, (*S*)-ibuprofen, and L-tryptophan, used in displacement experiments, were prepared in the mobile phase, i.e., a mixture of potassium phosphate buffer (67 mM, pH 7.0)–ACN (87:13 *v*/*v*). All solutions were stored at 4 °C. Probe solutions were added to the mobile phase at various concentrations ranging from 5 to 20 µM (5, 10, 15, and 20 µM for warfarin and L-tryptophan, and 5, 7.5, 10, 12.5, 15, and 20 µM for (*S*)-ibuprofen). These concentrations were selected based on previous displacement studies [19,28]. An aqueous buffer of a 67 mM solution of potassium phosphate was prepared by adjusting the pH to 7.0 with a solution of saturated NaOH. The aqueous buffer pH was controlled with a Crison^®^ BasiC 20 pH meter.

### 3.4. Bound Fraction Determination

Zonal analysis was the approach selected for the development of this work. The retention of the analyte is directly related to its interaction with the immobilized HSA, and can be described with Equation (1) [29,57].

(1) $$k=\frac{{(K}_{A1}n_{1}+\ldots +K_{An}n_{n})m_{L}}{V_{M}}$$

The retention factor (*k*) is related to the number of binding sites and to their corresponding equilibrium constants (*K_A_*). *V_M_* represents the void volume and mL the total moles of the analyte binding sites. Equation (2) was used to calculate the retention factor [58].

(2) $$k=\frac{t_{r}-t_{0}}{t_{0}}$$

*t_r_* corresponds to the retention time of the analyte and *t*_0_ to the dead time. The *t*_0_ was taken from each individual run and was considered to be equal to the solvent front [19].

One application of zonal elution in HPAC is to measure the binding affinity of a drug to an immobilized protein. In true equilibrium, *k* is associated with bound fraction (*b*) trough Equation (3) [29]

(3) $$k=\frac{b}{f}$$

where *f* represents the free fraction of analyte in solution. It is possible to rearrange Equation (3) to calculate %*b* using only *k*, as the sum of the free factions and the bound fraction must equal one. %*b* was calculated according to Equation (4) [29].

(4) $$\%b=\frac{k}{1+k}\times 100$$

### 3.5. Displacement Experiments

Equation (5) can be used to describe the impact of the competitor (*I*) on the retention time of each analyte (*A*) [32,58]

(5) $$\frac{1}{k}=\frac{V_{M}K_{I}[I]}{K_{A}m_{L}}+\frac{V_{M}}{K_{A}m_{L}}$$

where *K_I_* is the association equilibrium constant of *I* at the binding site of competition with *A*, [*I*] is the concentration of the competitor in the mobile phase, and *K_A_* is the association equilibrium constant of *A* at the binding site of competition with *I*.

If a direct competition between the analyte and the competing agent occurs, a straight line with a positive slope is observed in the plot of 1/*k* versus [*I*]; if allosteric competition between the analyte and the competing agent occurs, a straight line with a negative slope is observed in the plot of 1/*k* versus [*I*]; and if random variations are observed, no competition between the analyte and the competing agent occurs [29,59]. If competition is observed, the equilibrium association constant (*K_I_*) of the competitor in the binding site of the analyte is obtained with Equation (6) [28,54]

(6) $$\frac{Slope}{Intercept}=\frac{\left(\frac{V_{M}K_{I}}{K_{A}m_{L}}\right)}{\left(\frac{V_{M}}{K_{A}m_{L}}\right)}=K_{I}=\frac{1}{K_{D}}\;$$

where *K_D_* is the equilibrium dissociation constant of the competitor in the binding site of the analyte.

### 3.6. Computational

The X-ray crystal structure of HSA was downloaded from the Protein Data Bank (PDB code: 2BXG) [60]. The structures of the synthetic cannabinoids 5F-AMB, AB-PINACA, AMB-FUBINACA, AB-CHMINACA, and ADB-FUBINACA were modelled in Open Babel 3.0.0 and GaussView 5.0 (Gaussian, Inc., Wallingford, CT, USA) [61]. Docking calculations were conducted considering binding pockets to be essential for the interactions between the drugs and HSA, using AutoDock Vina 4 (Molecular Graphics Lab, CCSB, The Scripps Research Institute, La Jolla, CA, USA) [62].

In the docking studies, the synthetic cannabinoids (ligands) were considered flexible and the HSA receptor rigid. AutoDock Vina ran using an exhaustiveness of 8 and one grid box according to the interaction site, specifically site I of HSA. AutoDock Vina was the graphical interface used to build the grid box plugin from PyMOL. The box was centered in the center of mass of the selection encompassing the residues comprising the HSA site. The grid box was centered in X: 5.00; Y: −8.00; Z: 7.00 and had the dimensions (Å) of X: 20.6, Y: 17.6, and Z: 18.0. The conformations with the lowest binding affinities were investigated, encompassing up to nine different conformations. PyMOL version 2.3.0 (Schrödinger, New York, NY, USA) was used for visual inspection of results and graphical representations [63].

To measure the lipophilicity of all synthetic cannabinoids, the partition coefficient between two solvents, n-octanol and water (Log P_o/w_), was calculated using the Swiss ADME software [19], provided by the Swiss Institute of Bioinformatics (Lausanne, Switzerland) (http://www.swissadme.ch/).

## 4. Conclusions

In this study, HPAC with an HSA column was used to investigate the interaction between the synthetic cannabinoids, 5F-AMB, AB-PINACA, AMB-FUBINACA, AB-CHMINACA, ADB-FUBINACA, and FUBIMINA, and HSA. The results demonstrate that all analytes exhibited a high affinity for HSA, with binding percentages exceeding 98.7%. FUBIMINA, the most lipophilic compound, displays the highest binding affinity (%*b* = 99.9%), suggesting a correlation between HSA binding and lipophilicity.

Displacement studies revealed that 5F-AMB, AB-PINACA, AMB-FUBINACA, and AB-CHMINICA compete allosterically with warfarin for site I, while AB-CHMINACA also competes allosterically with L-tryptophan for site II. Notably, the binding affinity for all synthetic cannabinoids increased with a rising (*S*)-ibuprofen concentration, resulting in a lower free fraction of these compounds. Consequently, (*S*)-ibuprofen may serve as a promising candidate for exploring potential strategies to mitigate the harmful effects of synthetic cannabinoids. However, additional competition assays are needed to explore this possibility further, involving other experimental bioaffinity-based separative techniques (such as equilibrium dialysis, ultrafiltration, or ultracentrifugation), as well as in silico molecular dynamic simulations.

Docking scores confirmed the binding affinity of 5F-AMB, AB-PINACA, AMB-FUBINACA, and AB-CHMINACA for site I of HSA, and the most relevant interactions were identified.

These findings provide valuable insights into potential competition affecting HSA binding, which may ultimately influence the activity and toxicity of synthetic cannabinoids. Extrapolated to real-world scenarios, particularly when synthetic cannabinoids are consumed in uncontrolled high doses or alongside other drugs with high affinity to site I of HSA, this may be a serious issue. Competition for HSA binding could increase the unbound fraction of synthetic cannabinoids in the bloodstream, potentially leading to enhanced side effects, toxicity, or even overdose.

## Figures and Tables

**Table 1:** Retention factor (*k*), binding affinity (%*b*), theoretical Log P_o/w_, and molecular weight for the synthetic cannabinoids studied.

Compound	*k* ^[a]^	Bound Fraction (%*b*) ^[a]^	Log P_o/w_ ^[b]^	Molecular Weight (g/mol) ^[b]^
5F-AMB	87.30	98.9	3.40	363.43
AB-PINACA	79.11	98.7	2.58	330.42
AMB-FUBINACA	326.38	99.7	3.50	383.42
AB-CHMINACA	294.40	99.7	2.89	356.46
ADB-FUBINACA	619.87	99.8	3.10	382.43
FUBIMINA	968.23	99.9	3.67	360.42

^[a]^ Values at 100% aqueous buffer. ^[b]^ Values obtained from the SwissADME web tool [19] provided by the Swiss Institute of Bioinformatics.

**Table 2:** Binding profile for each synthetic cannabinoid at the HSA sites.

Synthetic Cannabinoid	HSA Site I	HSA Site II
5F-AMB	Binding occurs (competition with warfarin).	Binding does not occur (no competition with L-tryptophan). Allosterically interacts with (*S*)-ibuprofen.
AB-PINACA	Binding occurs (competition with warfarin).	Binding does not occur (no competition with L-tryptophan). Allosterically interacts with (*S*)-ibuprofen.
AMB-FUBINACA	Binding occurs (competition with warfarin).	Binding does not occur (no competition with L-tryptophan). Allosterically interacts with (*S*)-ibuprofen.
AB-CHMINACA	Binding occurs (competition with warfarin).	Binding occurs (competition with L-tryptophan). Allosterically interacts with (*S*)-ibuprofen.
ADB-FUBINACA	Binding does not occur (no competition with warfarin).	Binding does not occur (no competition with L-tryptophan). Allosterically interacts with (*S*)-ibuprofen.

**Table 3:** *K_I_* values obtained using displacement chromatography analysis. Values were determined by the slope and intercept of the plot of 1/*k* of the analyte vs. increasing competitor concentrations.

Synthetic Cannabinoid	*K_I_* Warfarin (M^−1^) (Lit. 3.3 × 10^5^ M^−1^) [55]	*K_I_* (S)-Ibuprofen (M^−1^) (Lit. 2.7 × 10^6^ M^−1^) [55]	*K_I_* L-Tryptophan (M^−1^) (Lit. 4.4 × 10^4^ M^−1^) [55]
5F-AMB	1.27 × 10^2^	6.0 × 10^1^	-
AB-PINACA	1.86 × 10^2^	6.8 × 10^1^	-
AMB-FUBINACA	1.94 × 10^2^	6.8 × 10^1^	-
AB-CHMINACA	2.1 × 10^2^	7.7 × 10^1^	3.3 × 10^1^
ADB-FUBINACA	-	7.0 × 10^1^	-

**Table 4:** Binding free energy for the top conformation of each synthetic cannabinoid and warfarin (site I-specific probe).

Synthetic Cannabinoid	Binding Free Energy, Site I (kcal/mol)	Amino Acid Residues
5F-AMB	−8.4	His242; Tyr150; Arg257
AB-PINACA	−8.2	Tyr150; Lys199; Glu292
AMB-FUBINACA	−9.6	His242; Tyr150; Arg257
AB-CHMINACA	−9.0	Lys199; Glu292; Tyr150
ADB-FUBINACA	−9.5	Tyr150; Arg257
Warfarin	−9.8	-

## Data Availability

Data are contained within the article and Supplementary Materials.

## Supplementary Materials

The following supporting information can be downloaded at: https://www.mdpi.com/article/10.3390/ph18040581/s1, Figures S1–S6: Chromatograms for each synthetic cannabinoid on a CHIRALPAK^®^ HSA column. Figure S7: Calibration curves for each synthetic cannabinoid in the presence of increasing percentages of the organic modifier acetonitrile (ACN), expressed as a plot of 1/*k* of the analyte versus percentage of ACN; Figures S8–S26: IR, ^1^H NMR, ^13^C NMR, UV, and HRMS spectra of all synthetic cannabinoids.
