# Supplementary material for: Binding Affinity of Synthetic Cannabinoids to Human Serum Albumin: Site Characterization and Interaction Insights
Source: Pharmaceuticals (Basel). 2025 Apr 16;18(4):581. doi: 10.3390/ph18040581 (PMC12030568; doi:10.3390/ph18040581)
Supplement: Supplementary file 1 [file pharmaceuticals-18-00581-s001.zip › pharmaceuticals-3590483-supplementary.pdf]

Supplementary Data

Binding Affinity of Synthetic Cannabinoids to Human Serum Albumin: Site Characterization and Interaction Insights

Rita M. G. Santos [1], Rita Lima [1,2], Sara Cravo [1,2], Pedro Alexandrino Fernandes [3], Fernando Remião [4], and Carla Fernandes [1,2*]

[1] Laboratório de Química Orgânica e Farmacêutica, Departamento de Ciências Químicas, Faculdade de Farmácia, Universidade do Porto, Departamento de Ciências Químicas, Faculdade de Farmácia, Universidade do Porto, Rua Jorge Viterbo Ferreira, 228, 4050-313 Porto, Portugal; anaritasantos7890@gmail.com (R. M. G. S.); ritaalexandralima@gmail.com (R.L.); scravo@ff.up.pt (S.C.); cfernandes@ff.up.pt (C.F.)

[2] Interdisciplinary Center for Marine and Environmental Research (CIIMAR), University of Porto, Terminal de Cruzeiros do Porto de Leixões, Avenida General Norton de Matos, s/n, 4450-208 Matosinhos, Portugal

[3] LAQV, REQUIMTE, Departamento de Química e Bioquímica, Faculdade de Ciências, Universidade do Porto, Rua do Campo Alegre, S/N, 4169-007 Porto, Portugal; pafernan@fc.up.pt (P.A.F)

[4] UCIBIO – Applied Molecular Biosciences Unit, Laboratory of Toxicology, Department of Biological Sciences, Faculty of Pharmacy, University of Porto, Rua Jorge Viterbo Ferreira, 228, 4050-313 Porto, Portugal; remiao@ff.up.pt (F.R.)

* Correspondence: cfernandes@ff.up.pt (C.F.)

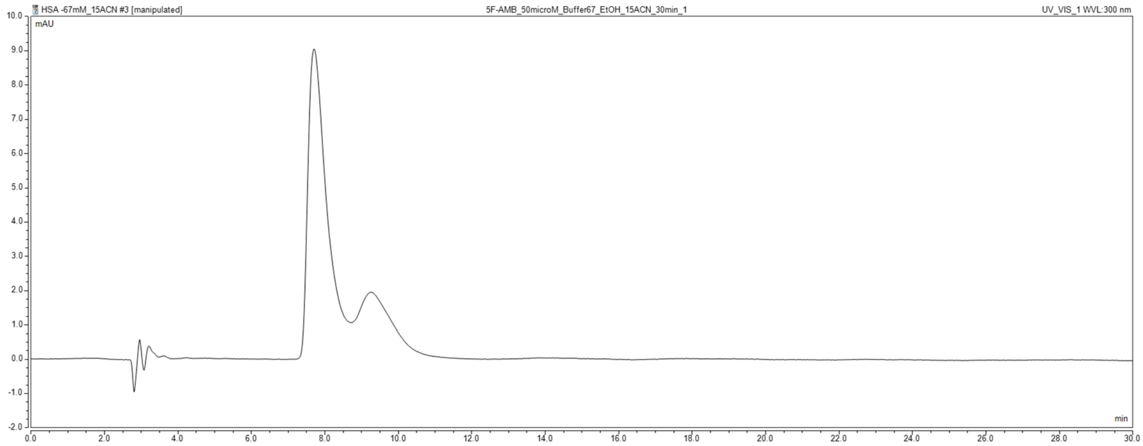

Figure S1. Chromatogram of 5F-AMB on a CHIRALPAK® HSA column. Flow rate: 0.5 mL/min. UV detection at 300 nm. Mobile phase: 67 mM potassium phosphate buffer solution (pH 7.0)–ACN (85:15 *v*/*v*). ACN: acetonitrile. The synthetic cannabinoid is present in equilibrium in its ionized form.

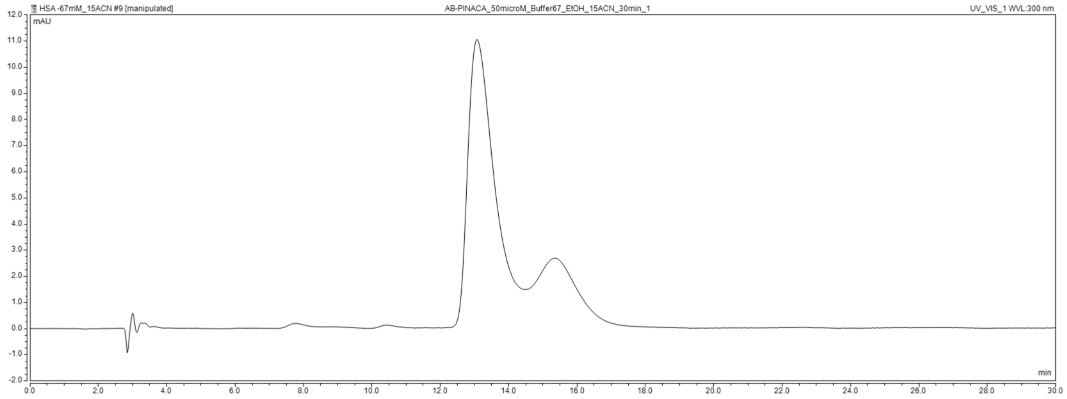

Figure S2. Chromatogram of AB-PINACA on a CHIRALPAK® HSA column. Flow rate: 0.5 mL/min. UV detection at 300 nm. Mobile phase: 67 mM potassium phosphate buffer solution (pH 7.0)–ACN (85:15 *v*/*v*). ACN: acetonitrile. The synthetic cannabinoid is present in equilibrium in its ionized form.

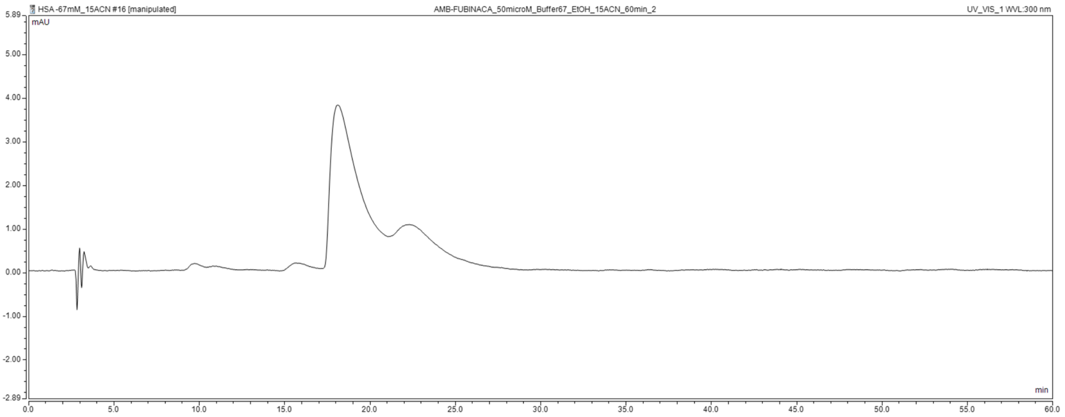

Figure S3. Chromatogram of AMB-FUBINACA on a CHIRALPAK® HSA column. Flow rate: 0.5 mL/min. UV detection at 300 nm. Mobile phase: 67 mM potassium phosphate buffer solution (pH 7.0)–ACN (85:15 *v*/*v*). ACN: acetonitrile. The synthetic cannabinoid is present in equilibrium in its ionized form.

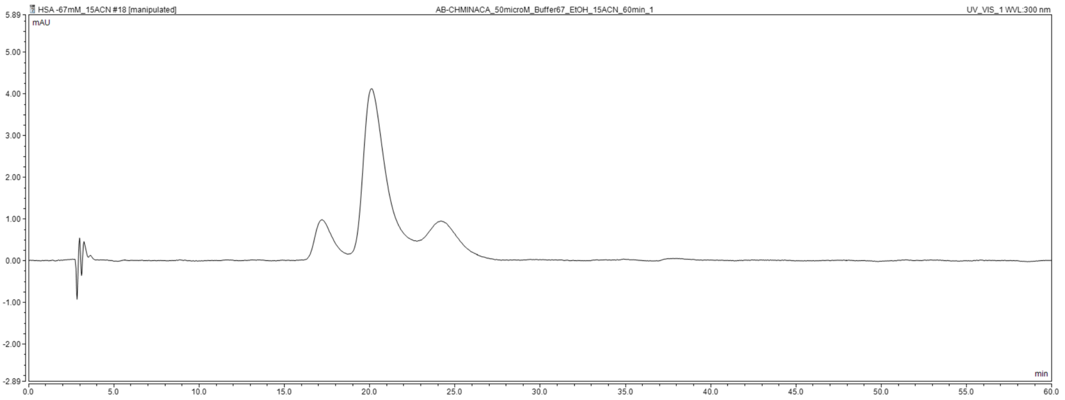

Figure S4. Chromatogram of AB-CHMINACA on a CHIRALPAK® HSA column. Flow rate: 0.5 mL/min. UV detection at 300 nm. Mobile phase: 67 mM potassium phosphate buffer solution (pH 7.0)–ACN (85:15 *v*/*v*). ACN: acetonitrile. The synthetic cannabinoid is present in equilibrium in its ionized form.

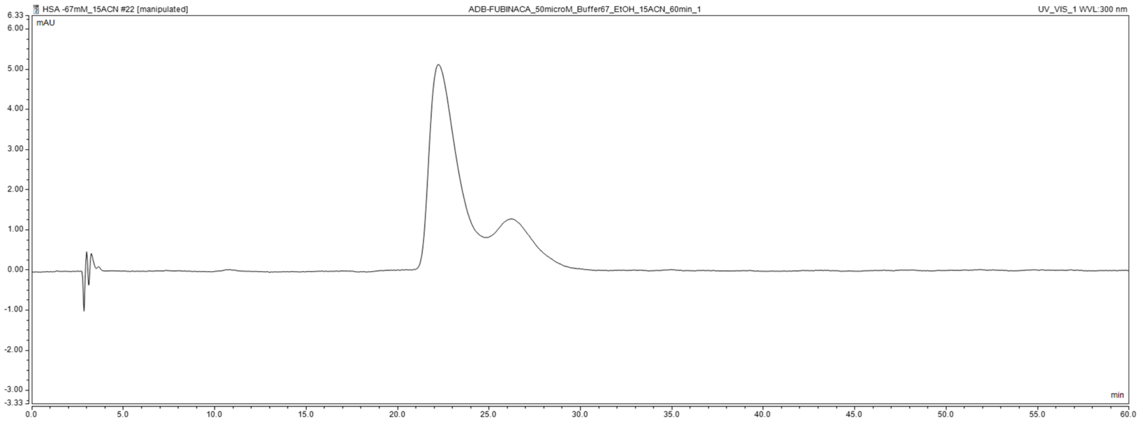

Figure S5. Chromatogram of ADB-FUBINACA on a CHIRALPAK® HSA column. Flow rate: 0.5 mL/min. UV detection at 300 nm. Mobile phase: 67 mM potassium phosphate buffer solution (pH 7.0)–ACN (85:15 *v*/*v*). ACN: acetonitrile. The synthetic cannabinoid is present in equilibrium in its ionized form.

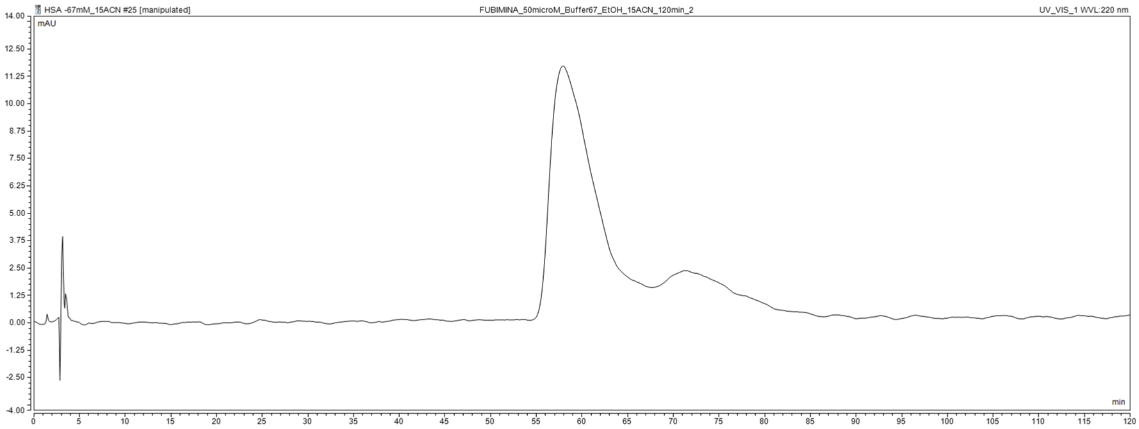

Figure S6. Chromatogram of FUBIMINA on a CHIRALPAK® HSA column. Flow rate: 0.5 mL/min. UV detection at 220 nm. Mobile phase: 67 mM potassium phosphate buffer solution (pH 7.0)–ACN (85:15 *v*/*v*). ACN: acetonitrile. The synthetic cannabinoid is present in equilibrium in its ionized form.

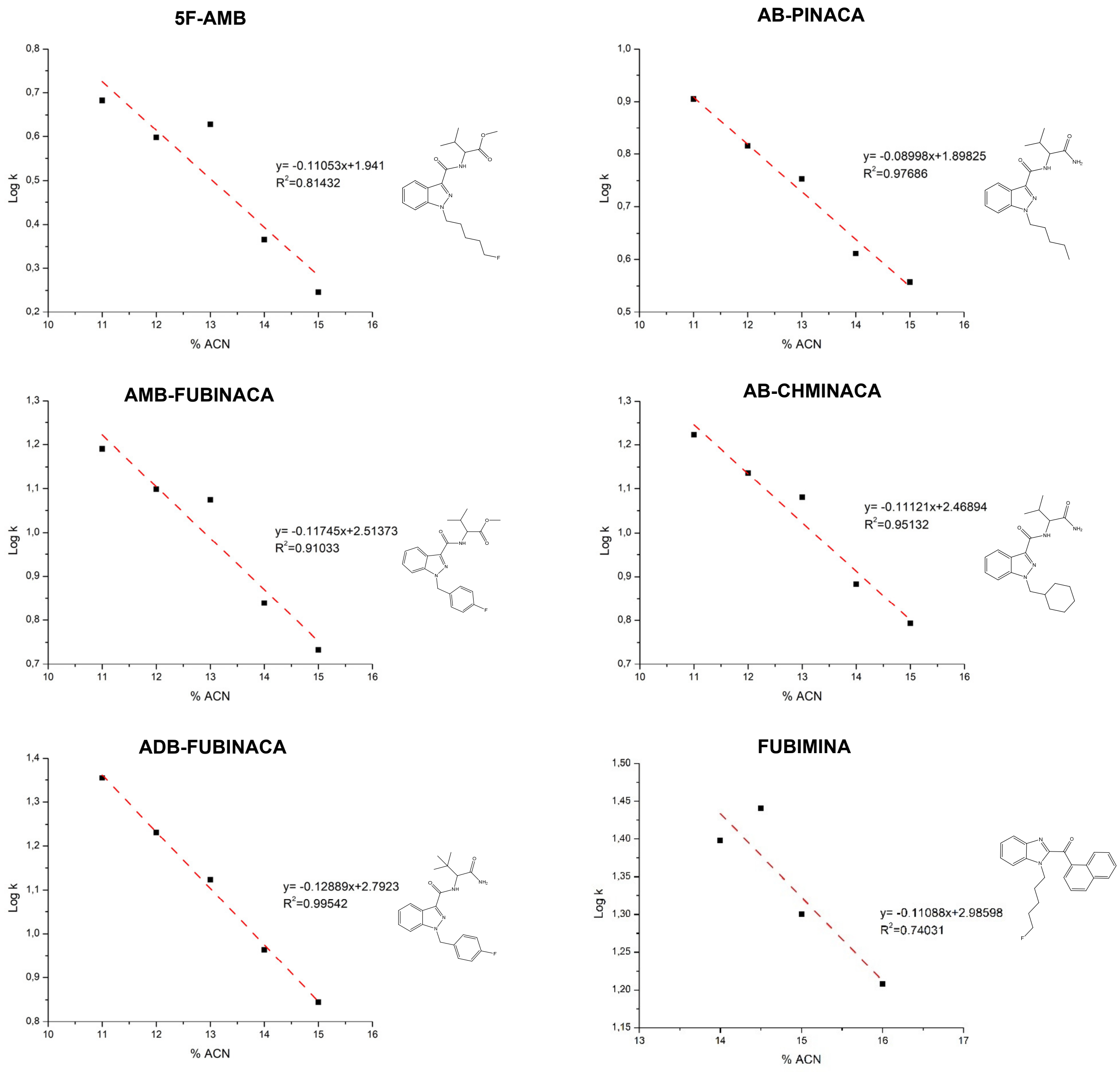

Figure S7. Calibration curves for each synthetic cannabinoid in the presence of increasing percentages of the organic modifier acetonitrile (ACN), expressed as a plot of 1/*k* of the analyte versus percentage of ACN.

IR, ^{1}H NMR, ^{13}C NMR, and HRMS data of synthetic cannabinoids

Infrared (IR) spectra were obtained using KBr microplates in a Fourier transform IR spectrometer Nicolet iS10 from (Thermo Fisher Scientifc Inc., Waltham, MA, USA) with a Smart OMNI Transmission accessory (Software OMNIC 8.3) (cm^{-1}). The ^{13}C and ^{1}H-NMR spectra were obtained from CEMUP (Centro de Materiais da Universidade do Porto), Porto, Portugal, on a Bruker Ascend III 400 instrument (^{1}H: 400.14 MHz; ^{13}C: 100.63MHz). Chemical shifts are expressed in ppm values to tetramethylsilane (TMS) as an internal reference, and coupling are reported in hertz (Hz). NMR spectra were taken in DMSO-d_6 at room temperature. Assignment abbreviations are the following: singlet (s), doublet (d), triplet (t), quartet (q), heptet (hept), multiplet (m), doublet of doublets (dd), doublet of triplets (dt), doublet of doublets of doublets (ddd), and broad (br). HRMS spectra were obtained from CEMUP, Porto, Portugal with a LTQ Orbitrap XL hybrid mass spectrometer (Thermo Fischer Scientific, Bremen, Germany). The limit of deviation between the calculated and observed m/z values for the product ions in HRMS is 10 ppm. UV–Vis spectra were obtained using a HITACHI UH5300 spectrophotometer (HITACHI, Ltd, Chiyoda, Tokyo, Japan).

5F-AMB

IR ν_{max} (cm^{-1}) (KBr): 3367.94, 2979.23-2875.35, 1737.77, 1653.14; ^{1}H NMR (400 MHz, DMSO-d_6) δ 8.13 (dt, *J* = 8.2, 1.1 Hz, 1H), 8.05 (d, *J* = 8.3 Hz, 1H), 7.80 (dt, *J* = 8.5, 1.0 Hz, 1H), 7.47 (ddd, *J* = 8.5, 6.9, 1.2 Hz, 1H), 7.28 (ddd, *J* = 7.9, 6.8, 0.9 Hz, 1H), 4.53 (t, *J* = 7.1 Hz, 2H), 4.49 – 4.41 (m, 2H), 4.35 (t, *J* = 6.0 Hz, 1H), 3.69 (s, 3H), 2.30 – 2.21 (m, 1H), 1.93 (p, *J* = 7.3 Hz, 2H), 1.75 – 1.61 (m, 2H), 1.40 – 1.32 (m, 2H), 0.96 (t, *J* = 6.4 Hz, 6H); ^{13}C NMR (101 MHz, DMSO-d_6) δ 171.97, 161.89, 140.56, 136.19, 126.66, 122.54, 122.04, 121.56, 110.45, 84.41, 82.80, 57.19, 51.84, 48.56, 29.88, 29.37, 29.18, 28.96, 22.03, 19.05, 18.59; HRMS (ESI^{+}) m/z calculated for [M] $C_{19}H_{26}FN_3O_3$ 363.19582, m/z observed for $[M]+K^{+}$ 402.15496, with a calculated error of -9.99ppm.

AB-PINACA

IR ν_{max} (cm^{-1}) (KBr): 3379.74, 3320.98-3179.34, 2957.51-2859.36, 1682.66; ^{1}H NMR (400 MHz, DMSO-d_6) δ 8.16 (dt, J = 8.2, 1.1 Hz, 1H), 7.78 (dd, J = 8.6, 1.0 Hz, 1H), 7.71 – 7.63 (m, 2H), 7.46 (ddd, J = 8.4, 6.9, 1.2 Hz, 1H), 7.28 (ddd, J = 7.9, 6.9, 0.9 Hz, 1H), 7.22 (d, J = 2.1 Hz, 1H), 4.50 (t, J = 7.1 Hz, 2H), 4.42 (dd, J = 9.1, 6.2 Hz, 1H), 2.16 – 2.03 (m, 1H), 1.87 (p, J = 7.2 Hz, 2H), 1.37 – 1.19 (m, 4H), 0.96 – 0.88 (m, 6H), 0.83 (t, J = 7.1 Hz, 3H); ^{13}C NMR (101 MHz, DMSO-d_6) δ 172.62, 161.32, 140.59, 136.35, 126.60, 122.45, 121.95, 121.64, 110.43, 56.73, 48.66, 31.26, 29.05, 28.27, 21.61, 19.35, 17.91, 13.80; HRMS (ESI^{+}) m/z calculated for

[M] $C_{18}H_{26}N_4O_2$ 330.20558, m/z observed for [M]+K^+ 369.16542, with a calculated error of -8.98ppm

AMB-FUBINACA

IR ν_{max} (cm^{-1}) (KBr): 3404.30, 3061.91-2874.21, 1737.61-1710.65, 1665.22; ^{1}H NMR (400 MHz, DMSO-d_6) δ 8.18 (d, *J* = 8.2 Hz, 1H), 8.14 (dt, *J* = 8.2, 1.0 Hz, 1H), 7.79 (dt, *J* = 8.6, 1.0 Hz, 1H), 7.46 (ddd, *J* = 8.4, 6.9, 1.1 Hz, 1H), 7.35 (ddd, *J* = 8.7, 5.7, 3.0 Hz, 2H), 7.29 (ddd, *J* = 7.9, 6.9, 0.8 Hz, 1H), 7.20 – 7.13 (m, 2H), 5.78 (s, 2H), 4.43 (dd, *J* = 8.3, 6.8 Hz, 1H), 3.69 (s, 3H), 2.25 (hept, *J* = 6.4 Hz, 1H), 0.96 (dd, *J* = 7.9, 6.8 Hz, 6H); ^{13}C NMR (101 MHz, DMSO-d_6) δ 171.95, 162.84, 161.83, 160.42, 140.51, 136.88, 132.97, 132.94, 129.50, 129.42, 127.00, 122.74, 122.39, 121.70, 115.59, 115.38, 110.57, 57.34, 51.83, 51.62, 29.82, 19.06, 18.71; HRMS (ESI^+) m/z calculated for [M] $C_{21}H_{22}FN_3O_3$ 383.16452, m/z observed for [M]+H+ 384.16938, with a calculated error of -6.29ppm. The most stable peak observed is 262.13725, possibly corresponding to the formation of the adduct $[2M+NH_4+2H]^{3+}$.

AB-CHMINACA

IR ν_{max} (cm^{-1}) (KBr): 3373.66, 3195.47, 2962.83-2851.88, 1693.90-1651.46; ^{1}H NMR (400 MHz, DMSO-d_6) δ 8.15 (dt, *J* = 8.1, 1.0 Hz, 1H), 7.77 (dt, *J* = 8.8, 0.9 Hz, 1H), 7.68 (s, 1H), 7.66 (d, *J* = 2.1 Hz, 1H), 7.44 (ddd, *J* = 8.4, 6.8, 1.1 Hz, 1H), 7.29 – 7.19 (m, 2H), 4.41 (dd, *J* = 9.0, 6.2 Hz, 1H), 4.34 (d, *J* = 7.2 Hz, 2H), 2.08 (dd, *J* = 7.5, 5.8 Hz, 1H), 1.93 (ddt, *J* = 11.0, 7.2, 3.6 Hz, 1H), 1.68 – 1.55 (m, 3H), 1.48 (s, 2H), 1.13 (d, *J* = 8.7 Hz, 3H), 1.09 – 1.01 (m, 2H), 0.91 (dd, *J* = 17.7, 6.8 Hz, 6H); ^{13}C NMR (101 MHz, DMSO-d_6) δ 172.61, 161.34, 141.19, 136.39, 126.59, 122.40, 121.78, 121.58, 110.65, 56.75, 54.54, 38.38, 31.27, 30.00, 29.97, 25.77, 25.09, 25.06, 19.35, 17.98; HRMS (ESI^+) m/z calculated for [M] $C_{20}H_{28}N_4O_2$ 356.22123, m/z observed for [M]+H^+ 357.22607, with a calculated error of -6.81ppm.

ADB-FUBINACA

IR ν_{max} (cm^{-1}) (KBr): 3402.01, 3199.96, 2965.15-2871.65, 1654.88; ^{1}H NMR (400 MHz, DMSO-d_6) δ 8.18 (dt, *J* = 8.1, 1.1 Hz, 1H), 7.79 (dt, *J* = 8.5, 0.9 Hz, 1H), 7.72 (d, *J* = 2.2 Hz, 1H), 7.61 (d, *J* = 9.7 Hz, 1H), 7.46 (ddd, *J* = 8.4, 7.0, 1.2 Hz, 1H), 7.35 – 7.29 (m, 3H), 7.27 (dd, *J* = 8.0, 1.5 Hz, 1H), 7.20 – 7.13 (m, 2H), 5.78 (s, 2H), 4.47 (d, *J* = 9.7 Hz, 1H), 0.99 (s, 9H); ^{13}C NMR (101 MHz, DMSO-d_6) δ 171.76, 162.87, 160.93, 160.44, 140.66, 136.97, 133.00, 129.52, 129.44, 127.08, 122.80, 122.25, 121.79, 115.65, 115.43, 110.66, 58.72, 51.65, 34.55, 26.63; HRMS (ESI^+) m/z calculated for [M] $C_{21}H_{23}FN_4O_2$ 382.18050, m/z observed for [M]+H^+ 383.18750, with a calculated error of -0.73ppm.

FUBIMINA

IR ν_{max} (cm^{-1}) (KBr): 3426.99, 3060.80-2861.69, 1655.15; ^{1}H NMR (400 MHz, DMSO-d_6) δ 8.24 – 8.18 (m, 2H), 8.11 – 8.05 (m, 1H), 7.95 (dd, *J* = 7.2, 1.2 Hz, 1H), 7.87 – 7.82 (m, 1H), 7.79

– 7.72 (m, 1H), 7.67 – 7.58 (m, 3H), 7.53 – 7.46 (m, 1H), 7.38 – 7.31 (m, 1H), 4.73 (t, *J* = 7.0 Hz, 2H), 4.54 – 4.46 (m, 1H), 4.41 – 4.33 (m, 1H), 1.96 (q, *J* = 7.4 Hz, 2H), 1.80 – 1.63 (m, 2H), 1.53 – 1.40 (m, 2H); ^{13}C NMR (101 MHz, DMSO-d_6) δ 189.13, 147.01, 141.32, 136.07, 134.92, 133.15, 132.23, 130.51, 130.23, 128.59, 127.64, 126.43, 125.86, 124.82, 124.51, 123.54, 121.45, 111.75, 84.42, 82.82, 44.80, 29.66, 29.51, 29.32, 22.15, 22.09; HRMS (ESI$^+$) m/z calculated for [M] $C_{23}H_{21}FN_2O$ 360.16379, m/z observed for [M]+H^+ 361.16802, with a calculated error of -8.44ppm.

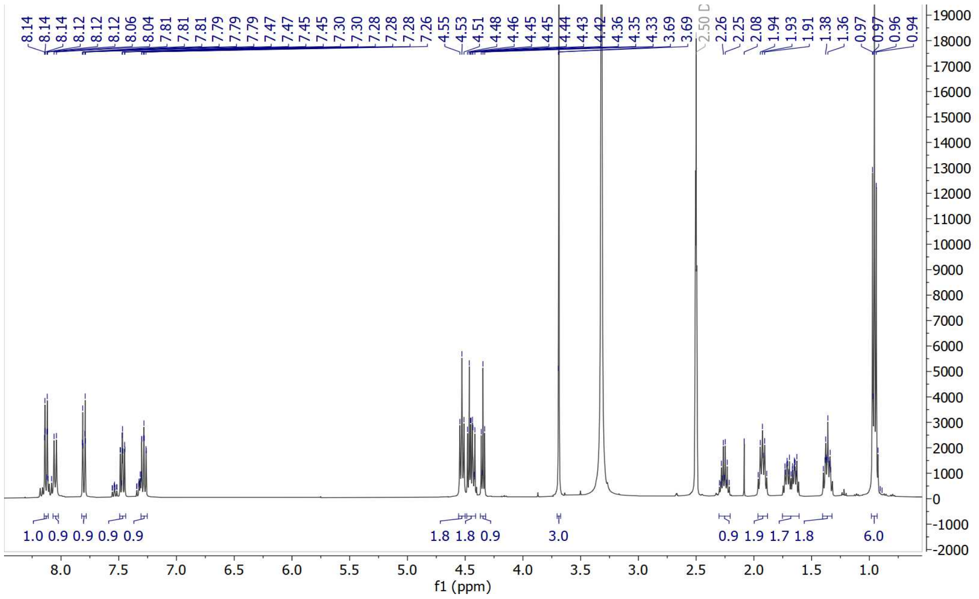

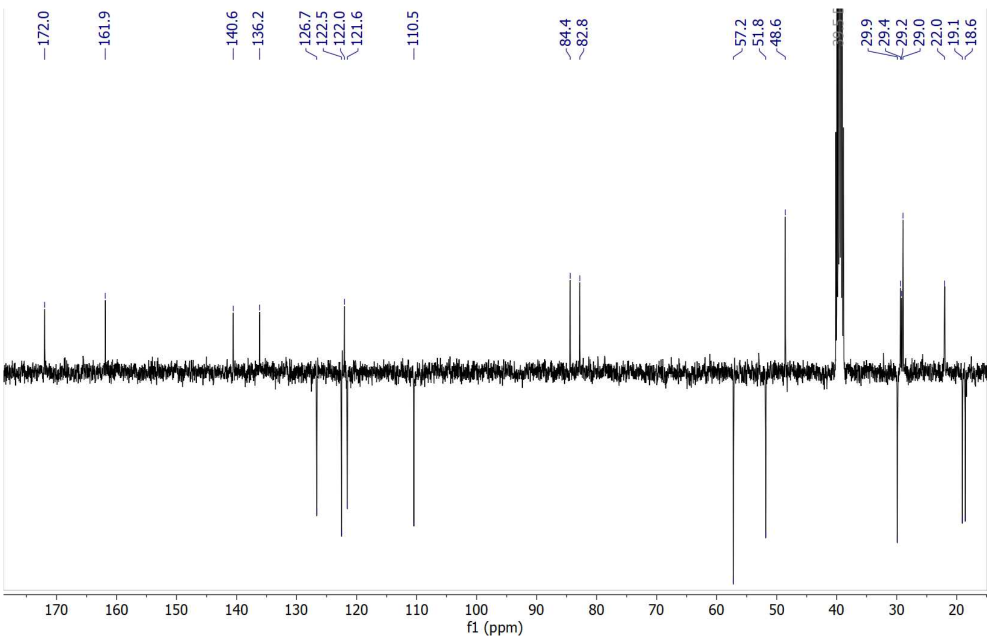

Figure S8. ^{1}H NMR (400 MHz, DMSO-d_6) and ^{13}C NMR (101 MHz, DMSO-d_6) for 5F-AMB.

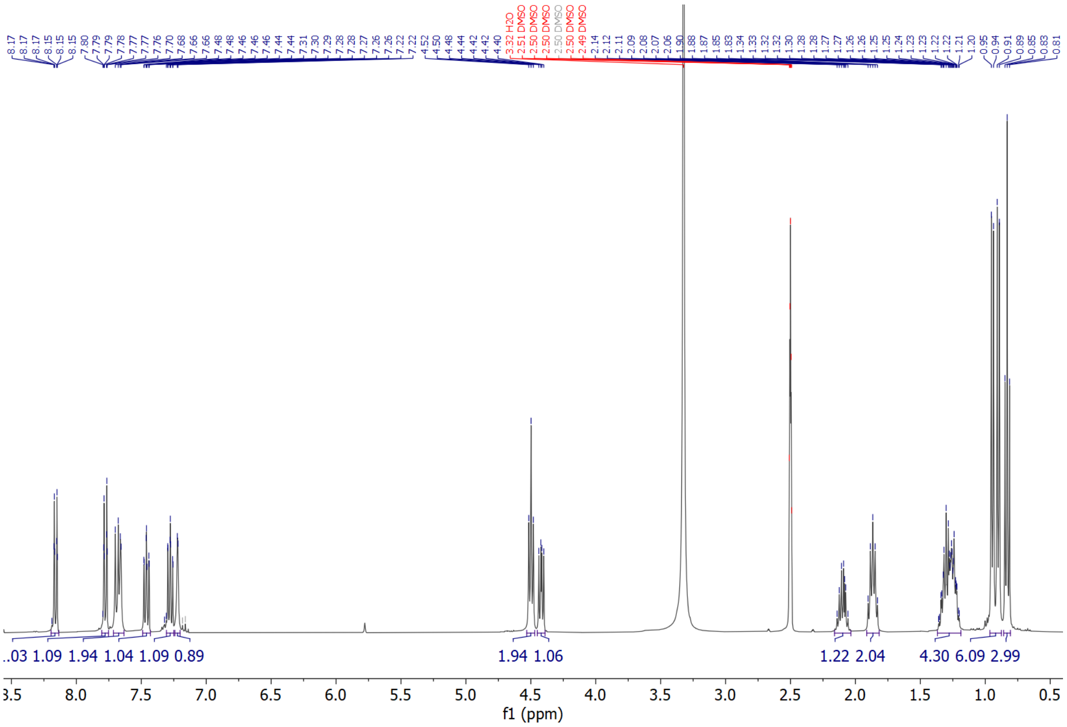

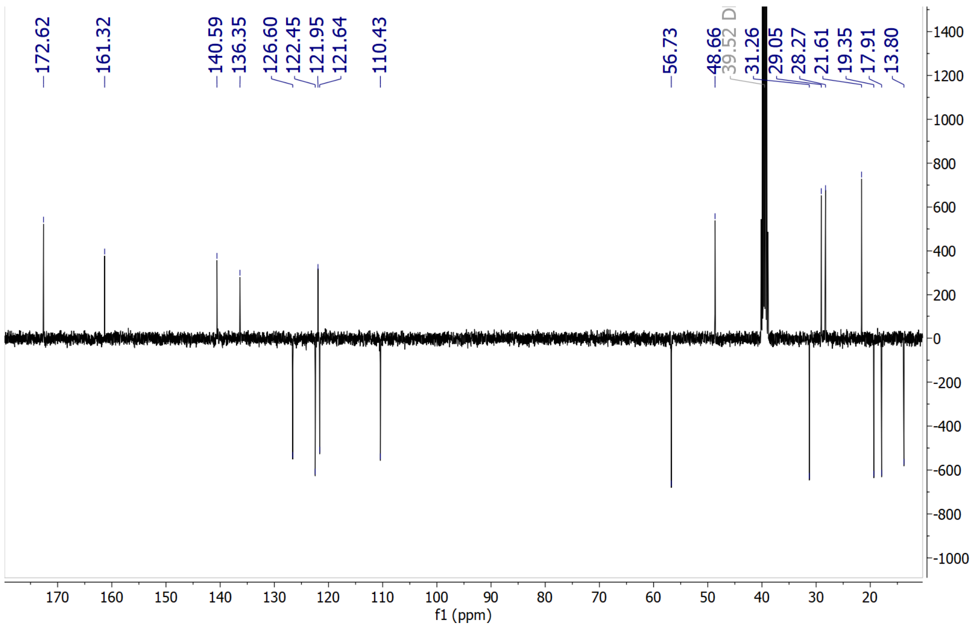

Figure S9. ^{1}H NMR (400 MHz, DMSO-d_6) and ^{13}C NMR (101 MHz, DMSO-d_6) for AB-PINACA.

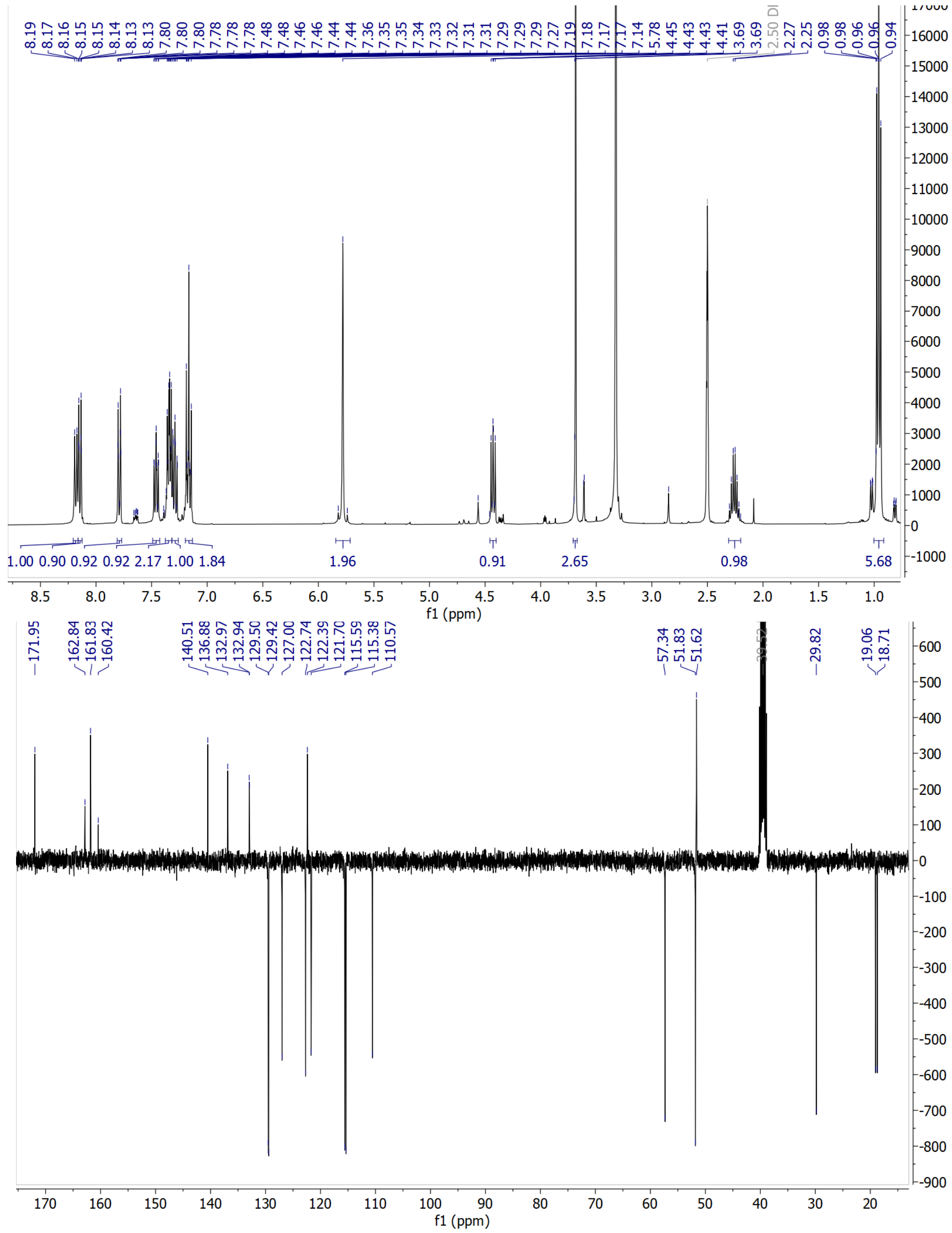

Figure S10. ^{1}H NMR (400 MHz, DMSO-d_6) and ^{13}C NMR (101 MHz, DMSO-d_6) for AMB-FUBINACA.

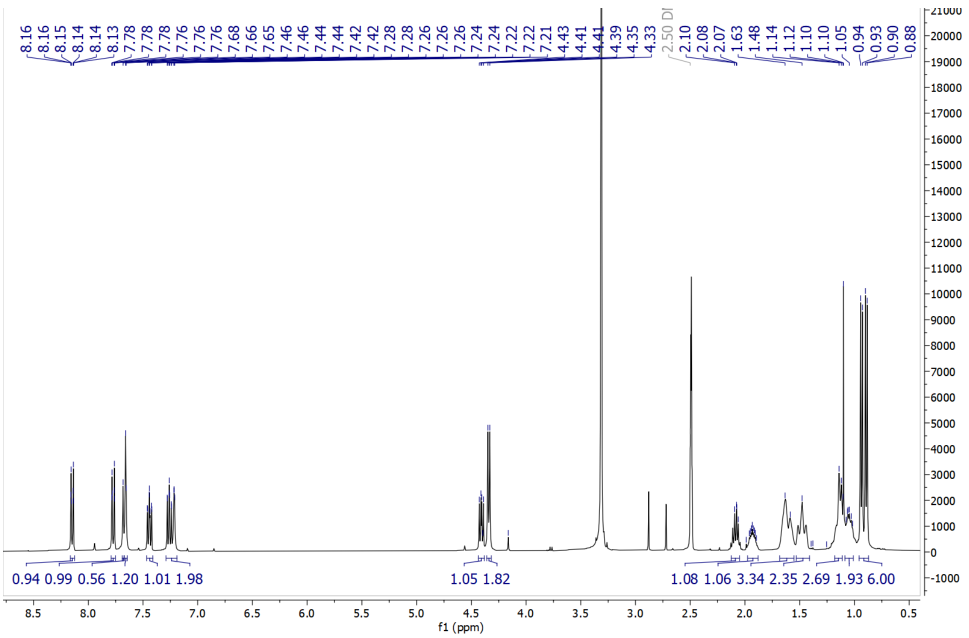

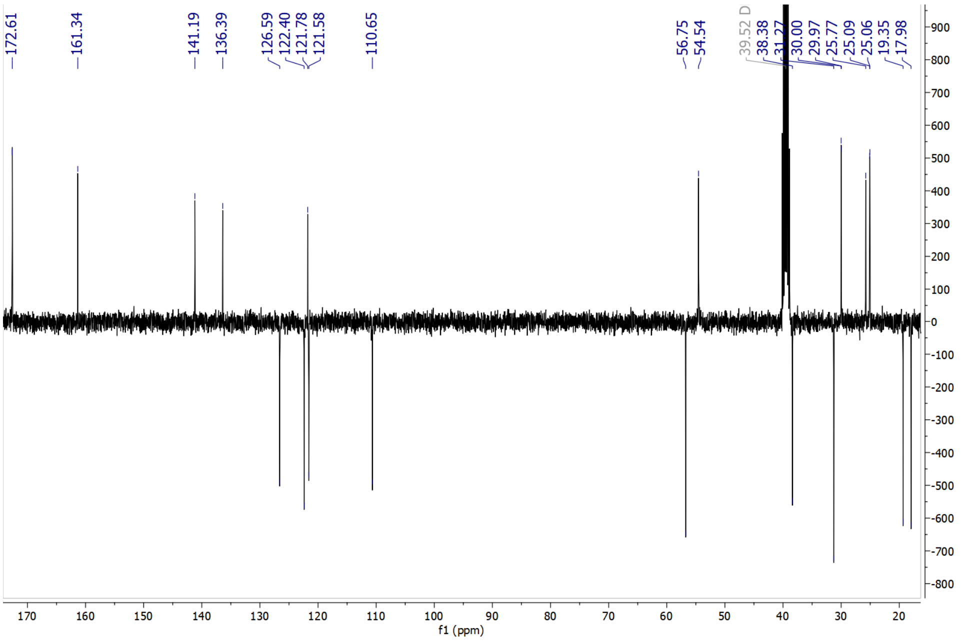

Figure S11. ^{1}H NMR (400 MHz, DMSO-d_6) and ^{13}C NMR (101 MHz, DMSO-d_6) for AB-CHMINACA.

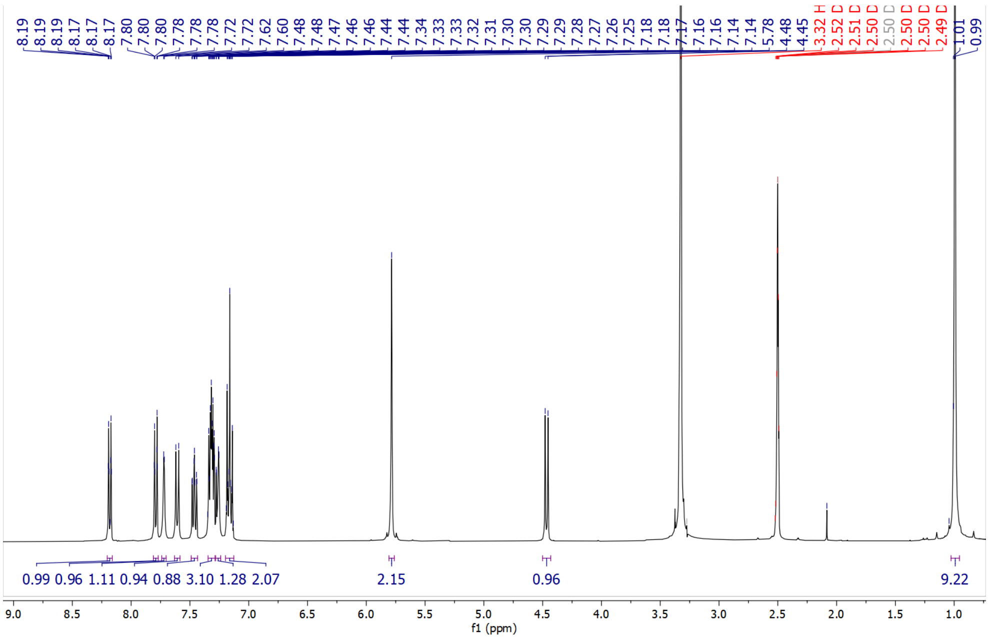

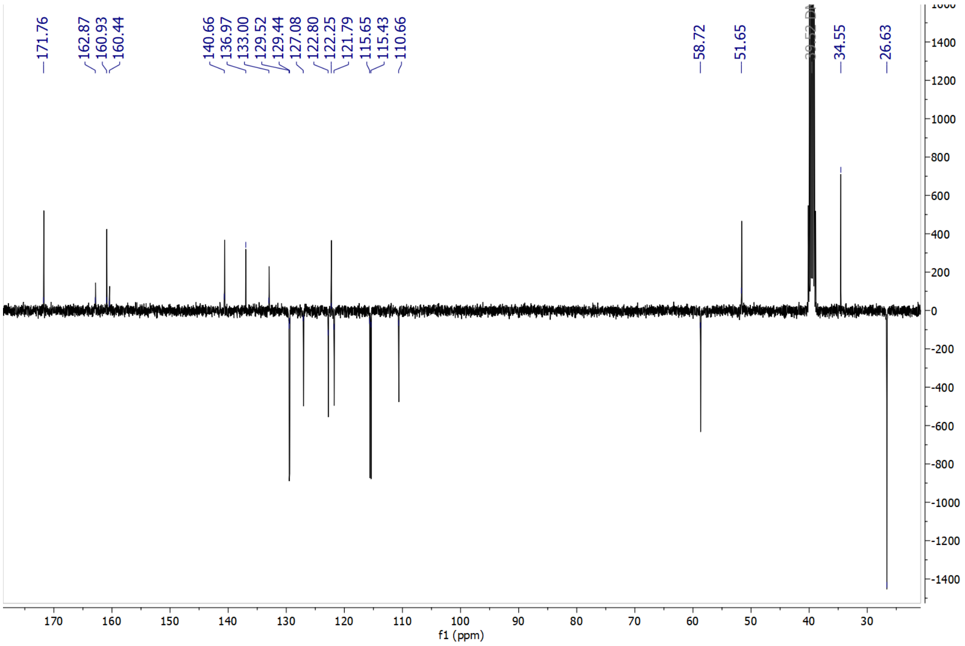

Figure S12. ^{1}H NMR (400 MHz, DMSO-d$_6$) and ^{13}C NMR (101 MHz, DMSO-d$_6$) for ADB-FUBINACA.

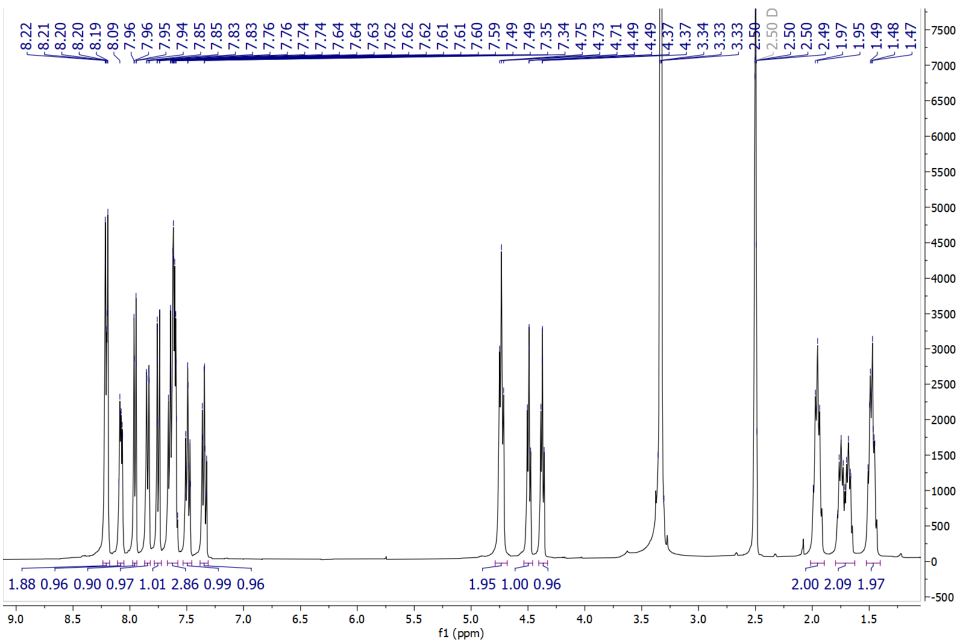

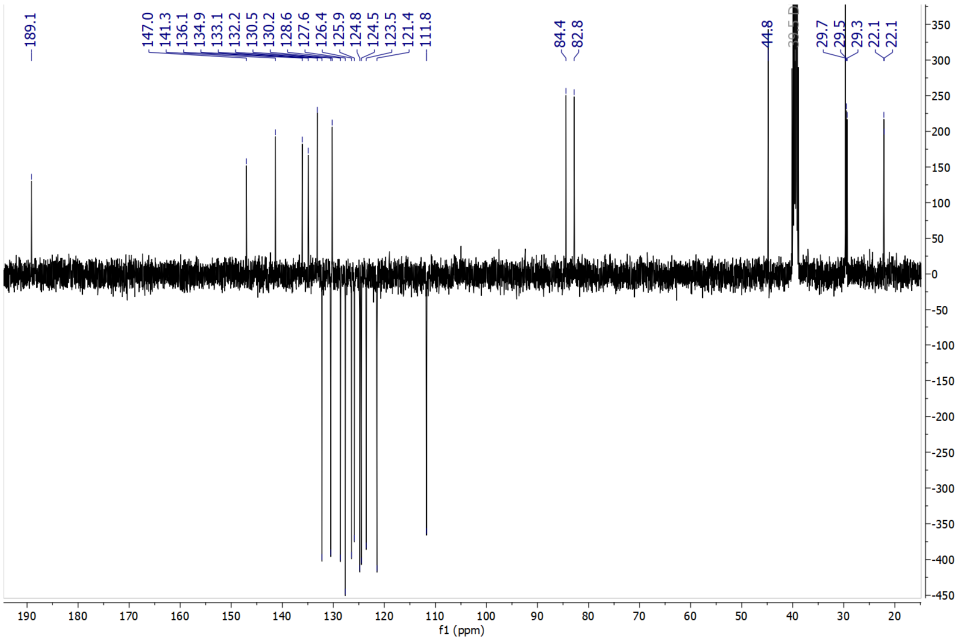

Figure S13. ^{1}H NMR (400 MHz, DMSO-d_6) and ^{13}C NMR (101 MHz, DMSO-d_6) for FUBIMINA.

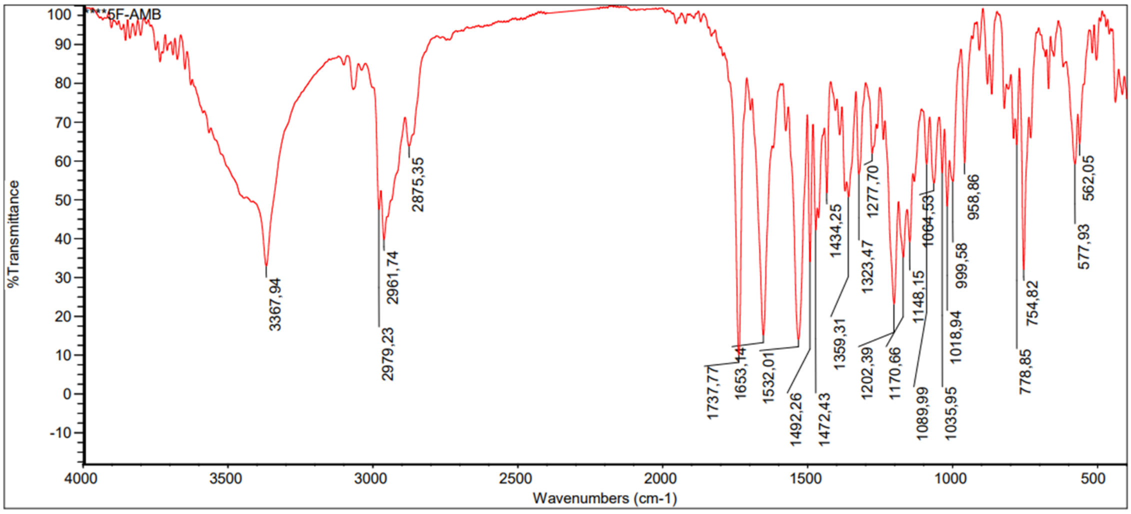

Figure S14. IR spectrum of 5F-AMB.

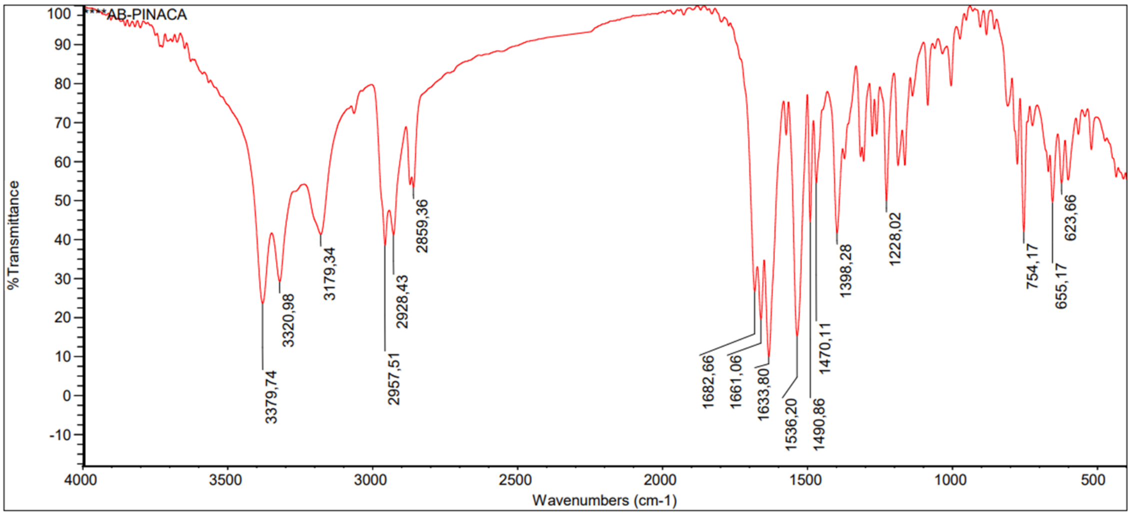

Figure S15. IR spectrum of AB-PINACA.

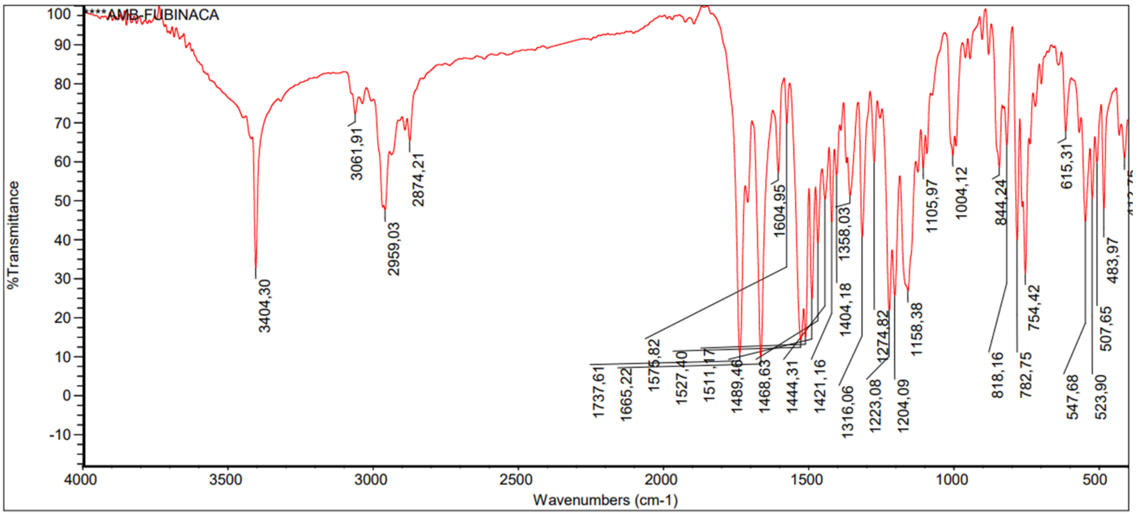

Figure S16. IR spectrum of AMB-FUBINACA.

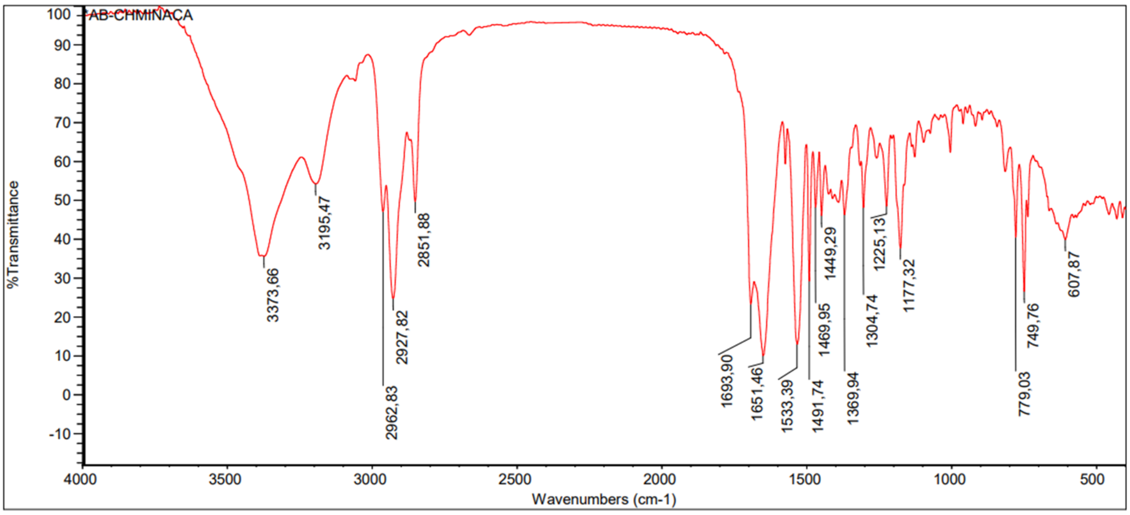

Figure S17. IR spectrum of AB-CHMINACA.

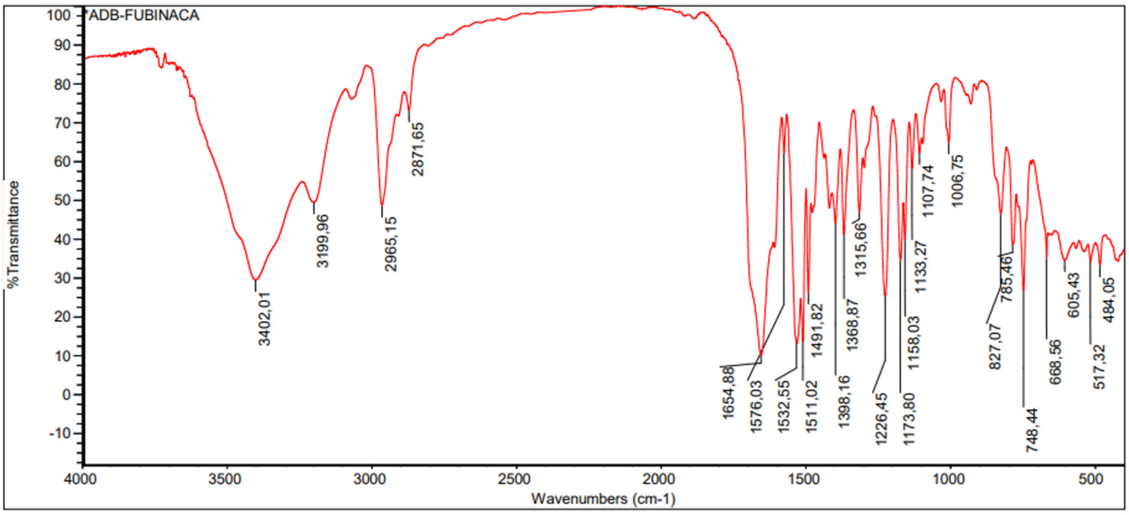

Figure S18. IR spectrum of ADB-FUBINACA.

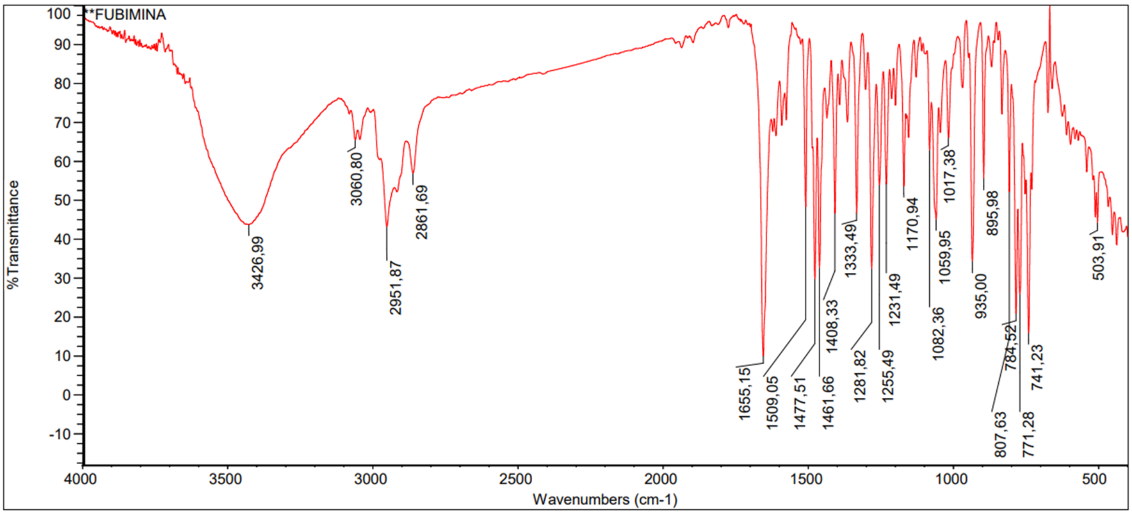

Figure S19. IR spectrum of FUBIMINA.

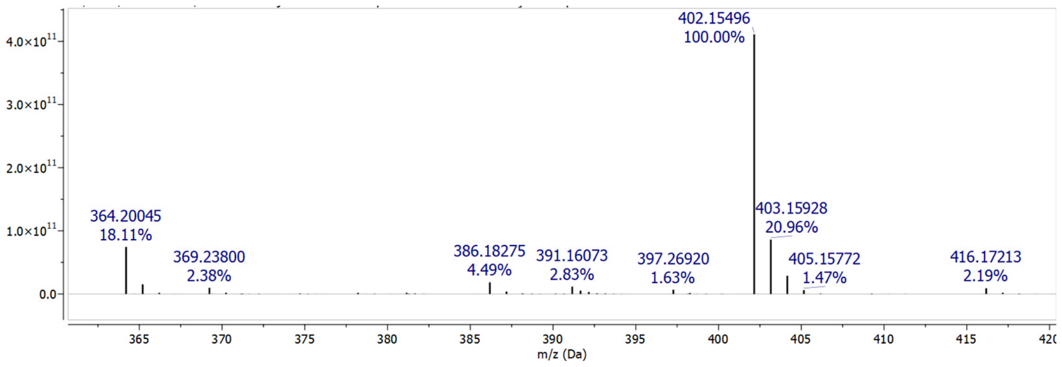

Figure S20. HRMS spectrum of 5F-AMB.

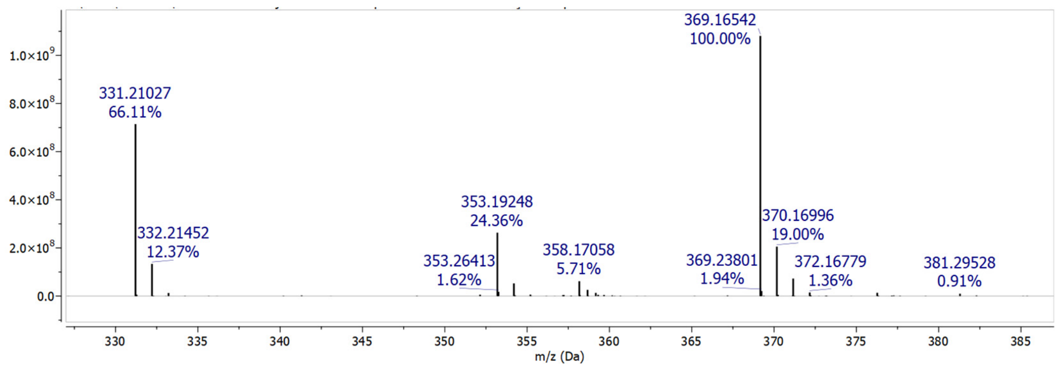

Figure S21. HRMS spectrum of AB-PINACA.

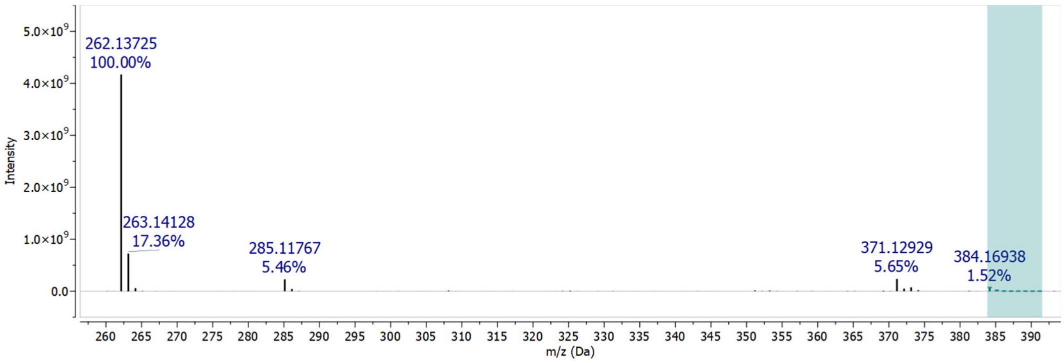

Figure S22. HRMS spectrum of AMB-FUBINACA.

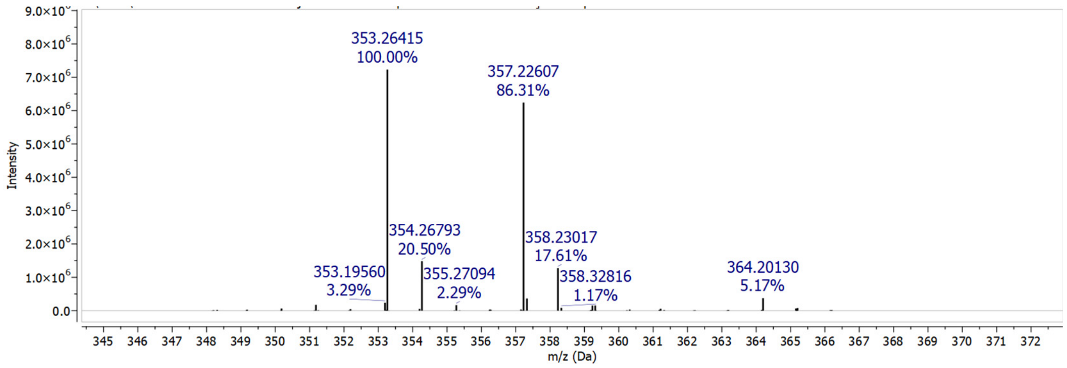

Figure S23. HRMS spectrum of AB-CHMINACA.

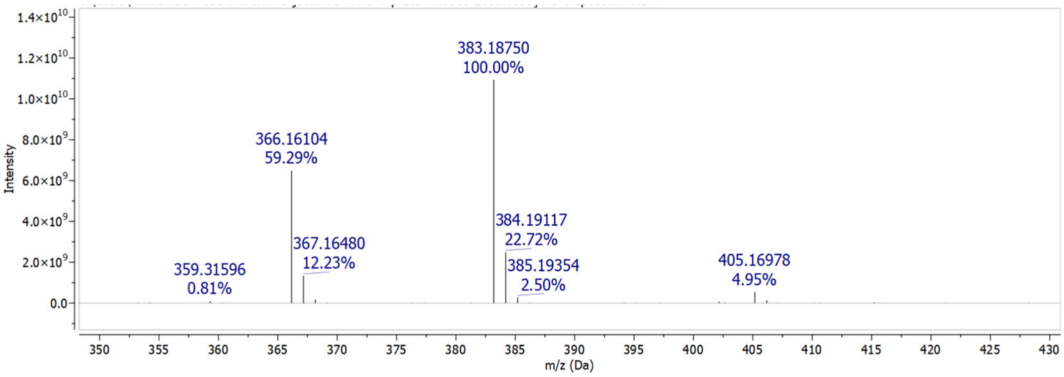

Figure S24. HRMS spectrum of ADB-FUBINACA.

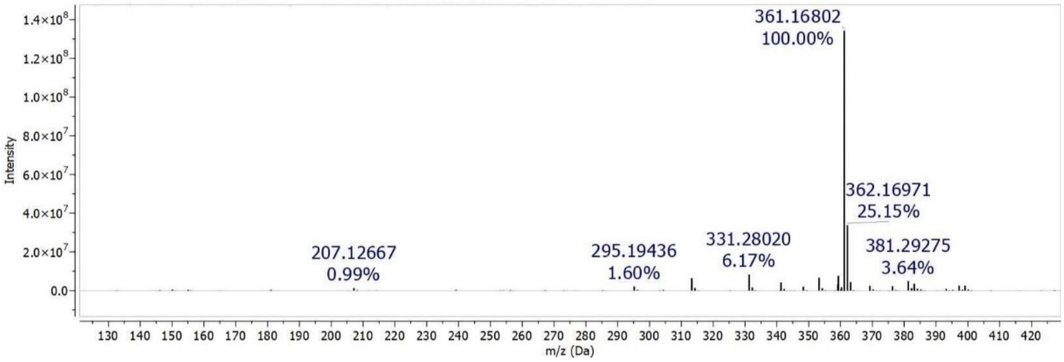

Figure S25. HRMS spectrum of FUBIMINA.

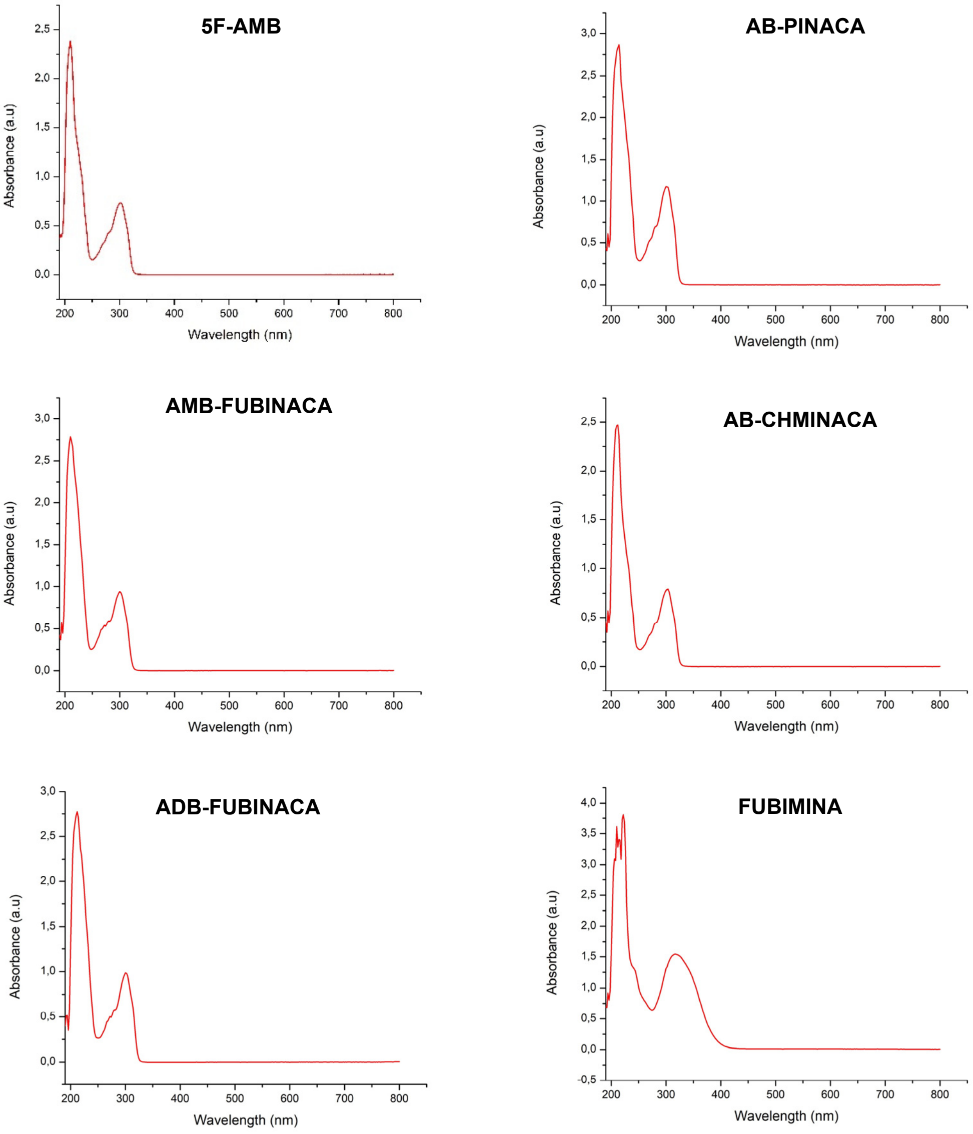

	Maximum absorption peaks (nm)		
**5F-AMB**	210	302	
**AB-PINACA**	214	300	
**AMB-FUBINACA**	210	300	
**ADB-FUBINACA**	212	300	
**AB-CHMINACA**	212	304	
**FUBIMINA**	210	222	316

Figure S26. Obtained UV spectra of the synthetic cannabinoids.
